# Bioinformatic Analyzes of the Association Between Upregulated Expression of *JUN* Gene *via APOBEC*-Induced *FLG* Gene Mutation and Prognosis of Cervical Cancer

**DOI:** 10.3389/fmed.2022.815450

**Published:** 2022-04-18

**Authors:** Huan Chen, Liyun Zhao, Jiaqiang Liu, Housheng Zhou, Xi Wang, Xiaoling Fang, Xiaomeng Xia

**Affiliations:** ^1^Department of Obstetrics and Gynecology, The Second XIANGYA Hospital of Central South University, Changsha, China; ^2^Laboratory Medicine Center, Zhu Zhou Hospital Affiliated to Xiangya School of Medicine, Central South University (CSU), Zhuzhou, China; ^3^Department of Obstetrics and Gynecology, Zhu Zhou Hospital Affiliated to Xiangya School of Medicine, CSU, Zhuzhou, China

**Keywords:** *APOBEC*, *FLG*, mutation, *JUN*, cervical cancer

## Abstract

Globally, cervical cancer (CC) is the most common malignant tumor of the female reproductive system and its incidence is only second after breast cancer. Although screening and advanced treatment strategies have improved the rates of survival, some patients with CC still die due to metastasis and drug resistance. It is considered that cancer is driven by somatic mutations, such as single nucleotide, small insertions/deletions, copy number, and structural variations, as well as epigenetic changes. Previous studies have shown that cervical intraepithelial neoplasia is associated with copy number variants (CNVs) and/or mutations in cancer-related genes. Further, CC is also related to genetic mutations. The present study analyzed the data on somatic mutations of cervical squamous cell carcinoma (CESC) in the Cancer Genome Atlas database. It was evident that the Apolipoprotein B mRNA editing enzyme-catalyzed polypeptide-like (*APOBEC*)-related mutation of the *FLG* gene can upregulate the expression of the *JUN* gene and ultimately lead to poor prognosis for patients with CC. Therefore, the findings of the current study provide a new direction for future treatment of CC.

## Introduction

Cervical cancer (CC) is the most common malignant tumor of the female reproductive system in the world and its incidence is only second after breast cancer (1). Cervical squamous cell carcinoma (CESC) is the primary subtype of CC, accounting for between 80 and 85% of all its diagnoses (2). According to the 2020 Global Cancer Statistics, CC ranks fourth in morbidity and mortality among women with cancer whereby there were 604,127 new cases and 41,831 deaths in the year (3). The highest global prevalence of CC occurs in developing countries (87%), of which it is the foremost form of gynecological cancer (4) whereas China accounts for 12% of the global incidence of CC and 11% of deaths (5). The CC screening program aims to reduce the incidence and mortality of the cancer (6). CC screening can detect abnormalities in the cells of the cervix, such as precancerous lesions, and early stages of CC (7). Screening for cervical cytology and human papilloma virus (HPV) is the most common method of CC screening (8). Although screening and advanced treatment strategies have improved the survival rates of patients with CC, some patients still die due to metastasis and drug resistance (9).

Copy number variants (CNVs) are genome rearrangements that lead to different copy numbers of genome segments, such as deletions, amplifications, and unbalanced translocations (10). Genomic structural variants are prevalent in the human genome (11) and represent an important source of genotype and phenotypic variation. CNVs usually play a functional role in regulating gene expression (12). Somatic mutations and other forms of genomic instabilities, such as single nucleotide variations (SNVs), small insertions/deletions (Indels), CNVs, structural variations, and epigenetic changes, can cause cancer (13).

Recently, the use of next-generation sequencing (NGS) has dramatically increased the number of somatic mutations detected in human cancers (14). For DNA-based analysis, Sanger sequencing remains the gold standard for the identification of somatic mutations (15). Currently, genomic studies have produced a wealth of data, including gene expression, copy number variation, and single nucleotide polymorphisms (SNPs) (16). The Cancer Genome Atlas (TCGA) is a large public database that includes 33 cancer types and matched clinical data (17). It provides a wide range of data types for patients with cancer, such as copy number variation, mRNA expression, and methylation.

The persistent infection of HPV has been recognized as an important cause of CC and precancerous lesions (18). However, only 10% of women infected with high-risk HPV develop precancerous lesions with less than 1% of the women progressing to CC (19). Although the occurrence and development of CC requires infection with certain types of HPV, other multiple factors are also involved but are not fully understood (20). Studies have shown that cervical intraepithelial neoplasia (CIN) is commonly associated with CNVs and/or mutations, such as KRAS and BRAF in cancer-related genes (21). Similarly, CC is also associated with genetic mutations (22, 23).

**Figure 1:**
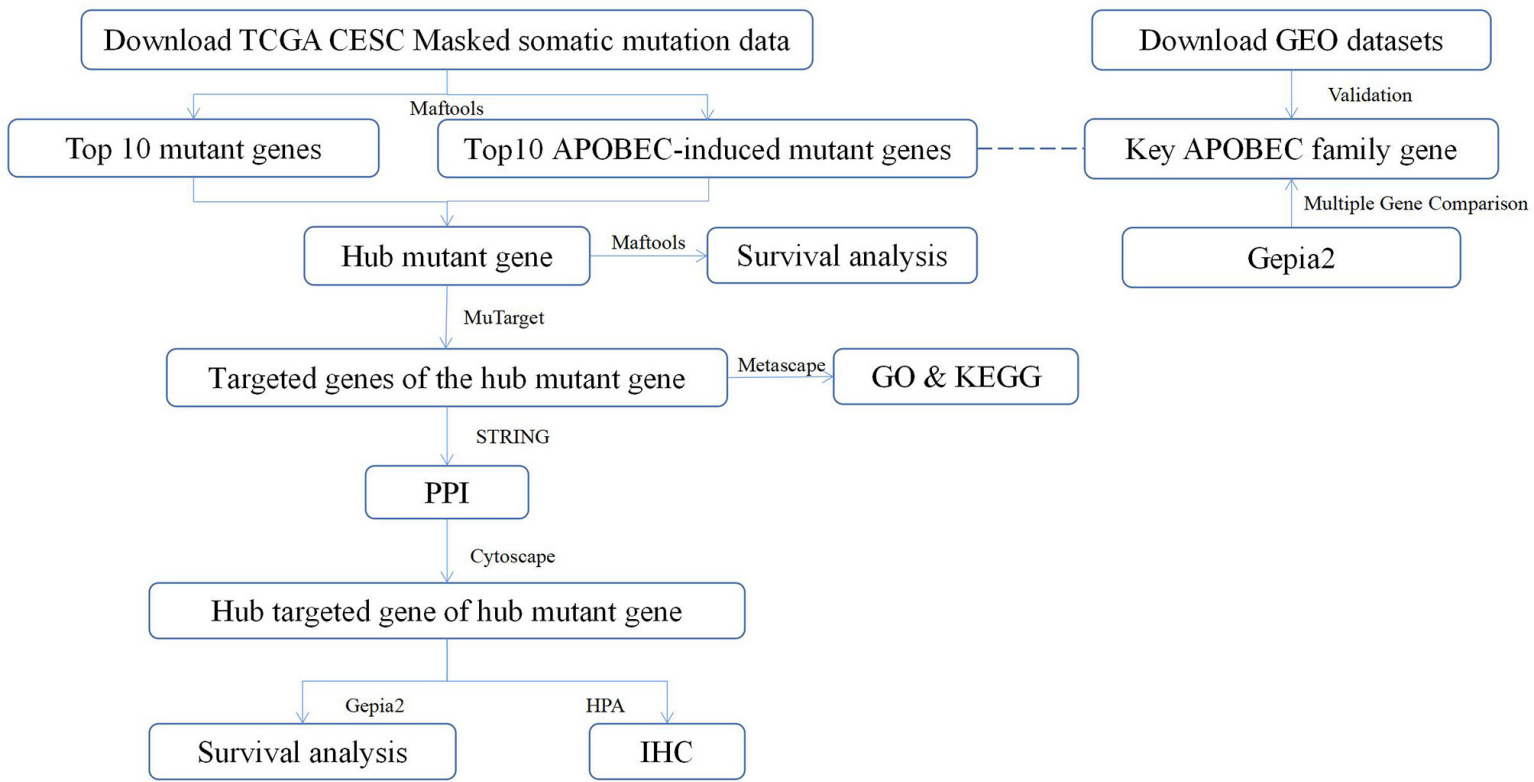
The workflow of the study. TCGA, The Cancer Genome Atlas; CESC, cervical squamous cell carcinoma; Maftools, a package in R language; MuTarget, an online platform, which could help us to identify the gene(s) showing altered expression in samples harboring a mutated input gene or identify mutations resulting in the expression change in the input gene (https://mutarget.com/); GO, gene oncology; KEGG, Kyoto Encyclopedia of Genes and Genomes; STRING, functional protein association networks platform (https://cn.string-db.org); PPI, protein–protein interaction; Cytoscape, an open-source software platform for dynamic, graphical visualization, manipulation, and analysis of networks; Gepia2, an online analysis platform based on TCGA database (http://gepia.cancer-pku.cn/); HPA, human protein atlas (https://www.proteinatlas.org) GEO, Gene Expression Omnibus; IHC, Immunohistochemistry.

Recently, the relationship between vaginal microbiology and CC has attracted attention of many researchers (24). Viral infections can induce the expression of Apolipoprotein B mRNA editing enzyme-catalyzed polypeptide-like (*APOBEC*) enzymes (25). Further, in addition to attacking viral genetic material, *APOBEC* enzymes can also attack human ssDNA, resulting in mutations that may contribute to cancer development (26). A previous study has reported that *APOBEC*-mediated mutations are the most significant contributor of the occurrence and development of CC (27). The present study analyzed the somatic mutation data of CESC in the TCGA database to evaluate the potential of *APOBEC*-associated mutated genes and target genes that are closely related to CC as well as provide new ideas for the future treatment of CC. The workflow of the study is shown in Figure 1.

## Materials and Methods

### Date Download and Procession

The TCGA is the largest database of genetic information on cancer (https://portal.gdc.cancer.gov/). Masked somatic mutations and associated clinicopathological data of patients with CC were downloaded from the TCGA database on 6 April 2021 for use in the current study. The samples included 302 cancer tissues and 8 adjacent tissue samples. The data were obtained, analyzed, and visualized using the “maftools” in the R software package (28). The Gene Expression Omnibus (GEO) datasets (GSE63514, GES6791, and GSE63678) obtained from the GEO database (http://www.ncbi.nlm.nih.gov/geo) were used as validation datasets for *APOBEC* family genes. These three datasets contain 24 normal cervix and 28 CC tissues; 5 normal cervix and 5 CC tissues; 8 normal cervix and 20 CC tissues, respectively.

### Identification of *APOBEC*-Induced Mutant Genes and Hub Mutant Genes

*APOBEC*-induced mutations are more common in solid tumors and are mainly associated with C>T transition events occurring in the TCW motif. Therefore, the present study analyzed the differences in mutation patterns between *APOBEC*-enriched and non-*APOBEC*-enriched samples using plotApobecDiff in the maftools package. Then, we overlapped the top 10 mutant genes and the top 10 *APOBEC*-induced mutant genes in the samples.

### Relationship Between Hub Mutant Genes and Survival

In the present study, Kaplan–Meier analysis was performed using the mafSurvive function in the Maftools package to investigate the prognostic value of hub mutant genes in CC.

### Identifying the Targeted Genes of Hub Mutant Genes

MuTarget (https://mutarget.com/) is an online platform, which can help in the identification of gene(s) showing altered expression in samples harboring a mutated input gene or mutations resulting in expression change in the input gene (29). The platform was used to identify the targeted genes associated with hub mutant genes whereby all target genes with *p* < 0.05 and mean fold change >1.44 were exported.

### Identification of the Hub Targeted Gene of Hub Mutant Genes

The targeted gene upregulation of the hub mutant gene was analyzed for protein–protein interaction (PPI) using online analysis website STRING (https://string-db.org/) (30), and the results were then entered into Cytoscape software (31) to calculate the hub targeted gene of hub mutant genes by using the Mcode plugin.

### Gene Oncology Analysis and Kyoto Encyclopedia of Genes and Genomes Pathway Enrichment

The gene oncology (GO) analysis and Kyoto Encyclopedia of Genes and Genomes (KEGG) pathway enrichment of the targeted genes of the hub mutant genes were carried out in the current study using Metascape (http://metascape.ncibi.org/).

### Identification of key *APOBEC* Family Genes

The current study analyzed the differential expression of *APOBEC* family genes using the online analysis tool—gepia2 (32) and the *APOBEC* family genes were validated in three independent datasets.

### Relationship Between the Hub Targeted Gene of Hub Mutant Genes and Survival

The present study analyzed the relationship between the hub targeted gene of hub mutant genes and overall survival of patients with CC using gepia2, an online analysis tool (32).

### Validation of the Hub Targeted Gene of Hub Mutant Genes

Protein expression of the hub targeted gene of hub mutant genes was validated by the Human Protein Atlas (https://www.proteinatlas.org/) (33).

## Results

### SNP Mutation and CNV Results in CESC Samples

**Figure 2:**
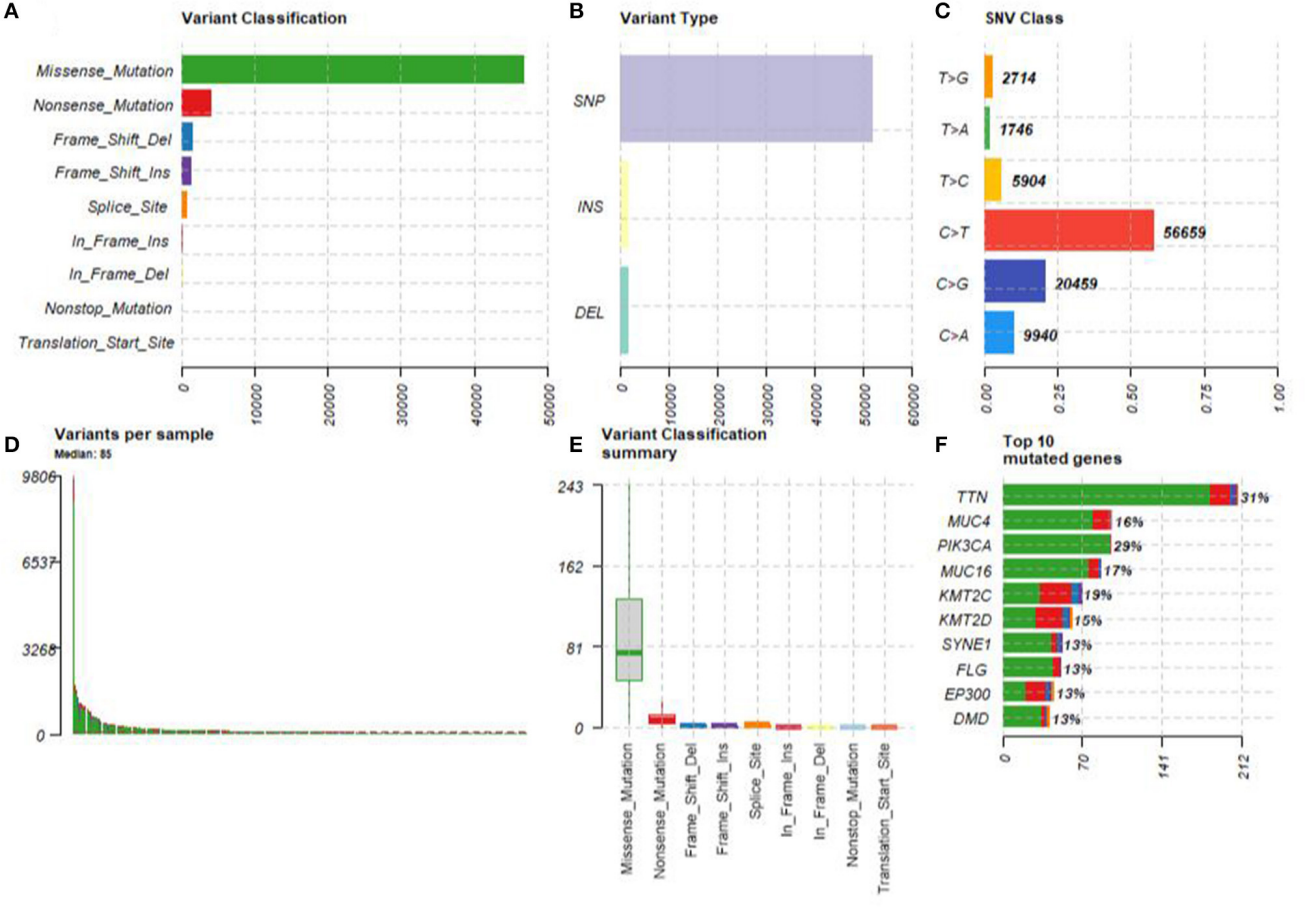
The summary of the TCGA CESC maf file. **(A)** The most common mutation was missense mutation, followed by nonsense mutation, frame shift del, frame shift ins, splice site, in-frame ins, in-frame del, and translation start site. **(B)** The most common variant type was single nucleotide polymorphism (SNP), followed by INS and DEL. **(C)** The most common SNV variant was C>T, followed by C>G, C>A, T>C, T>G, and T>A. **(D)** The number of variants per sample was 85. **(E)** The median missense mutation per sample was 74, followed by nonsense mutation, frame shift del, frame shift ins, splice site, in-frame ins, in-frame del, and translation start site. **(F)** The top 10 mutant genes were TNN, MUC4, PIK3CA, MUC16, KMT2C, KMT2D, SYNE1, FLG, EP300, and DMD.

**Figure 3:**
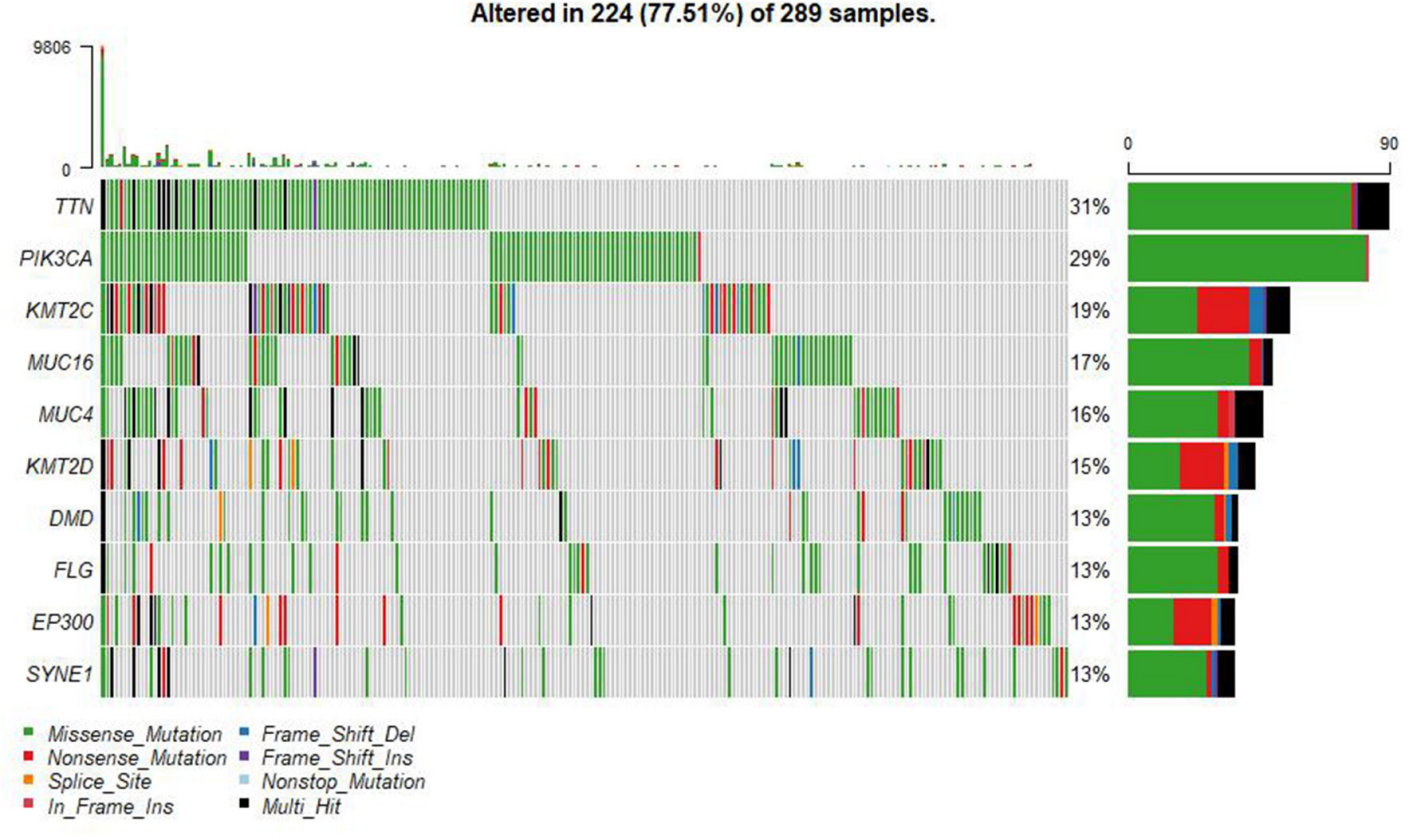
Summary of the TCGA CESC mutations. There were 55,768 mutations in all 289 samples. The top 10 mutant genes were TNN, MUC4, PIK3CA, MUC16, KMT2C, KMT2D, SYNE1, FLG, EP300, and DMD.

A summary of the TCGA CESC maf file in the present study is presented in Figure 2. After combining the mutation data with the corresponding clinical data, only 289 samples remained for follow-up analysis. In total, there were 55,768 mutations in all 289 samples. It was noted that the most common mutation in the CESC samples was missense mutation, followed by non-sense mutation, frame shift del, frame shift ins, splice site, in-frame ins, in-frame del, and translation start site. Result of the present study showed that the most common variant type was SNP, followed by INS and DEL. Further, it was noted that the most common SNV variant was C>T, followed by C>G, C>A, T>C, T>G, and T>A, whereas the number of variants per sample was 85 and the median missense mutation per sample was 74. In addition, the results of the current study found that the top 10 mutant genes were *TNN, MUC4, PIK3CA, MUC16, KMT2C, KMT2D, SYNE1, FLG, EP300*, and *DMD* and the mutations were detected in 224 out of the 289 samples (Figure 3).

### Identification of *APOBEC*-Induced Mutant Genes and Hub Mutant Genes

**Figure 4:**
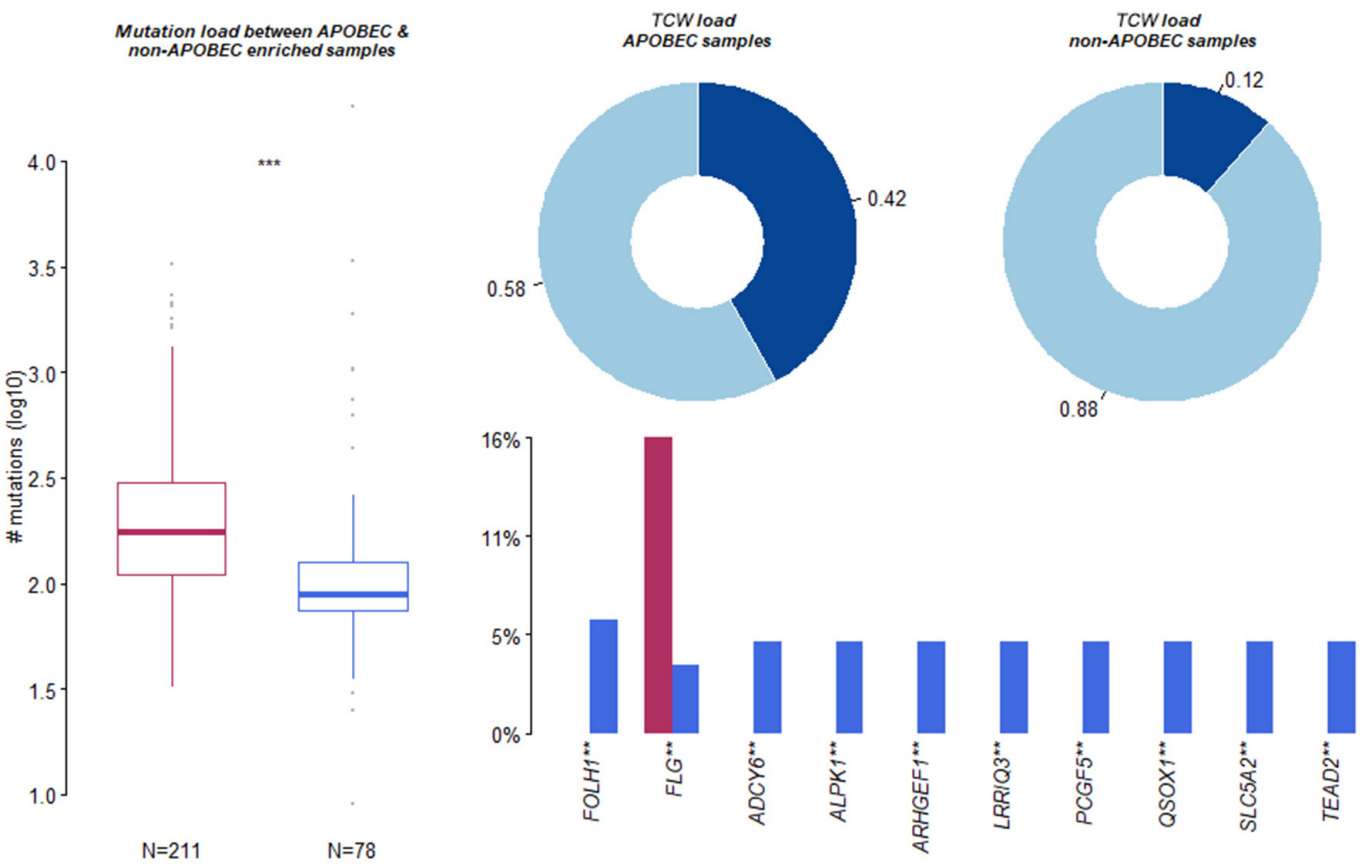
Summary of Apolipoprotein B mRNA editing enzyme-catalyzed polypeptide-like (APOBEC)-induced mutation. Apolipoprotein B mRNA editing enzyme-catalyzed polypeptide-like -enriched and non-enriched sample sizes were 211 and 78, respectively. The top 10 of 192 differentially APOBEC-induced mutant genes were FOLH1, FLG, ADCY6, ALPK1, ARHGEF1, LRRIQ3, PCGF5, QSOX1, SLC5A2, and TEAD2. **, *** means significant difference.

The present study used the maftools package to enrich C>T mutations occurring within TCW motifs on all C>T mutations in a given sample. Further, the C>T mutations were compared with background cytosines and TCWs occurring within 20 bp of the mutated base. In addition, the *APOBEC-*enriched and non-enriched sample sizes were 211 and 78, respectively (Figure 4). The top 10 of 192 differentially *APOBEC-*related mutant genes were *FOLH1, FLG, ADCY6, ALPK1, ARHGEF1, LRRIQ3, PCGF5, QSOX1, SLC5A2*, and *TEAD2*. Further, it was found that *FLG* was the hub mutant gene from the overlapped top 10 mutant genes and the top 10 *APOBEC*-related mutant genes.

### Identification of the Targeted Genes of the Hub Mutant Gene

A total of 124 genes with altered expression associated with *FLG* gene mutations were identified through muTarget website analysis. It was found that among the 124 genes, there were 99 downregulated genes and 25 upregulated genes.

### Identification of the Hub Targeted Gene of the Hub Mutant Gene

A PPI network analysis constructed the interaction networks for the upregulated targeted gene of hub mutant genes. It was found that *JUN* was the hub targeted gene among the hub mutant genes identified in the current study using the Mcode plugin.

### GO Analysis and KEGG Pathway Enrichment

**Figure 5:**
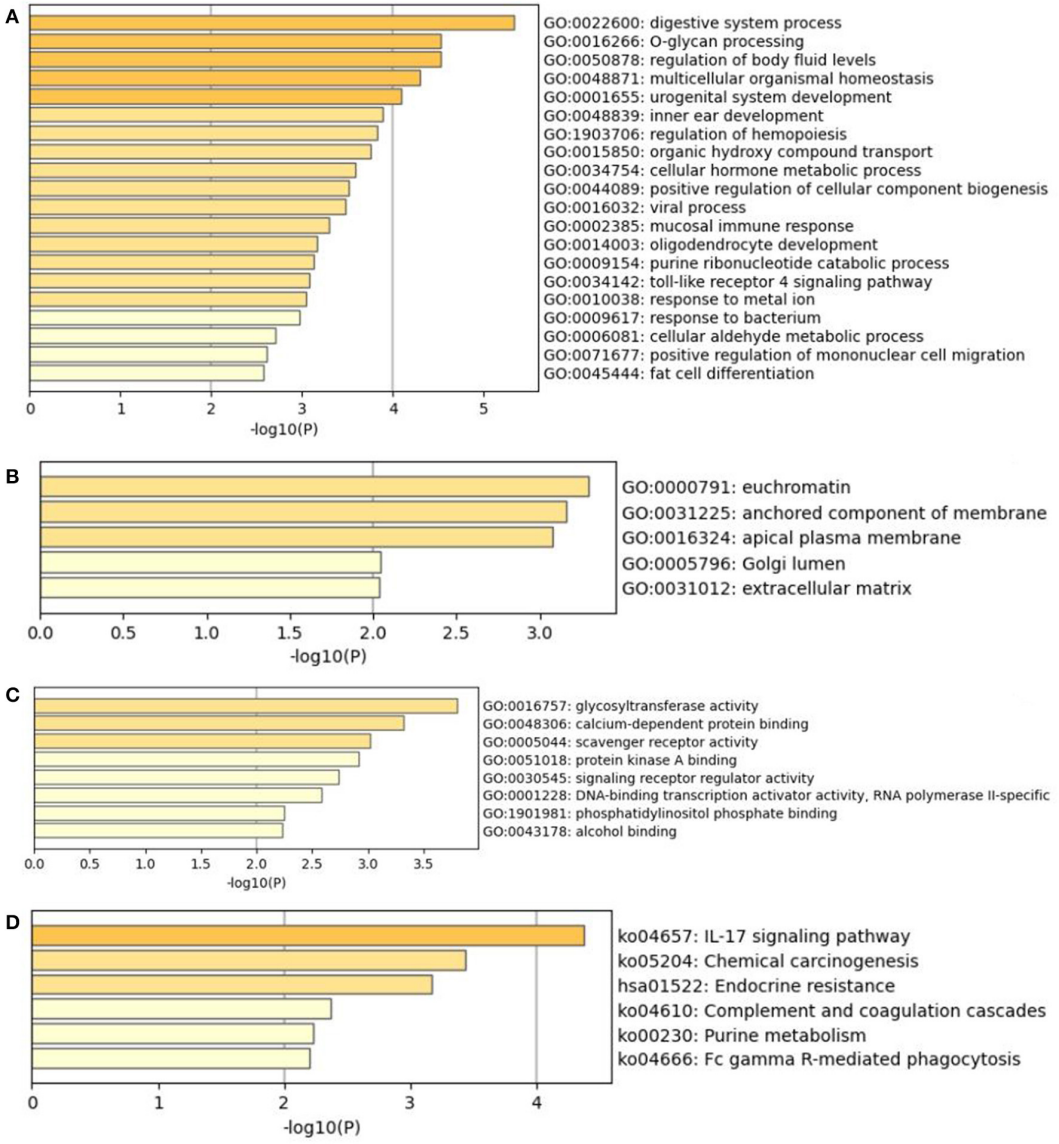
The GO analysis and KEGG pathway enrichment of hub mutant gene's targeted genes. **(A)** Biological processes (GO-BP). **(B)** Cellular components (GO-CC). **(C)** Molecular functions (GO-MF). **(D)** KEGG pathway of hub mutant gene's targeted genes.

The result of GO analysis and KEGG pathway enrichment in the current study is presented in Figure 5. GO analysis showed that 54 terms were significantly over represented (17 MF, 29 BP, and 8 CC). GO terms that *JUN* was involved in were DNA-binding transcription activator activity, response to drug, regulation of leukocyte differentiation, response to cAMP, and viral gene expression. Kyoto Encyclopedia of Genes and Genomes (KEGG) pathway analysis revealed that six pathways were significantly enriched. Moreover, the KEGG pathways that *JUN* was involved in were the IL-17 signaling pathway and endocrine resistance.

### Identification of *APOBEC3B* as a Key *APOBEC* Family Gene

**Figure 6:**
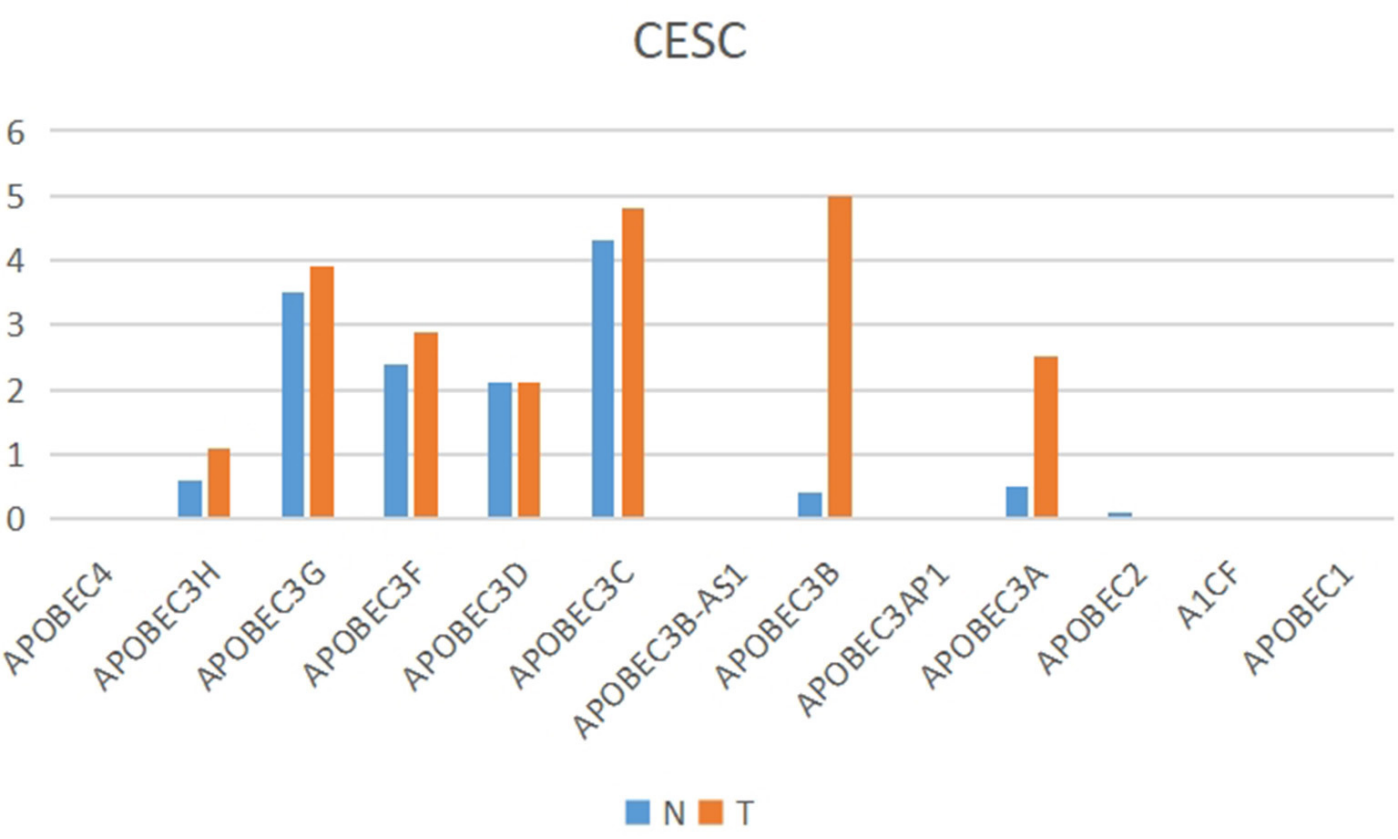
Multiple gene expression analysis of the APOBEC family in CESC. Among the APOBEC gene family, the expression of APOBEC3A and APOBEC3B was significantly higher in tumor tissues than in normal tissues.

**Figure 7:**
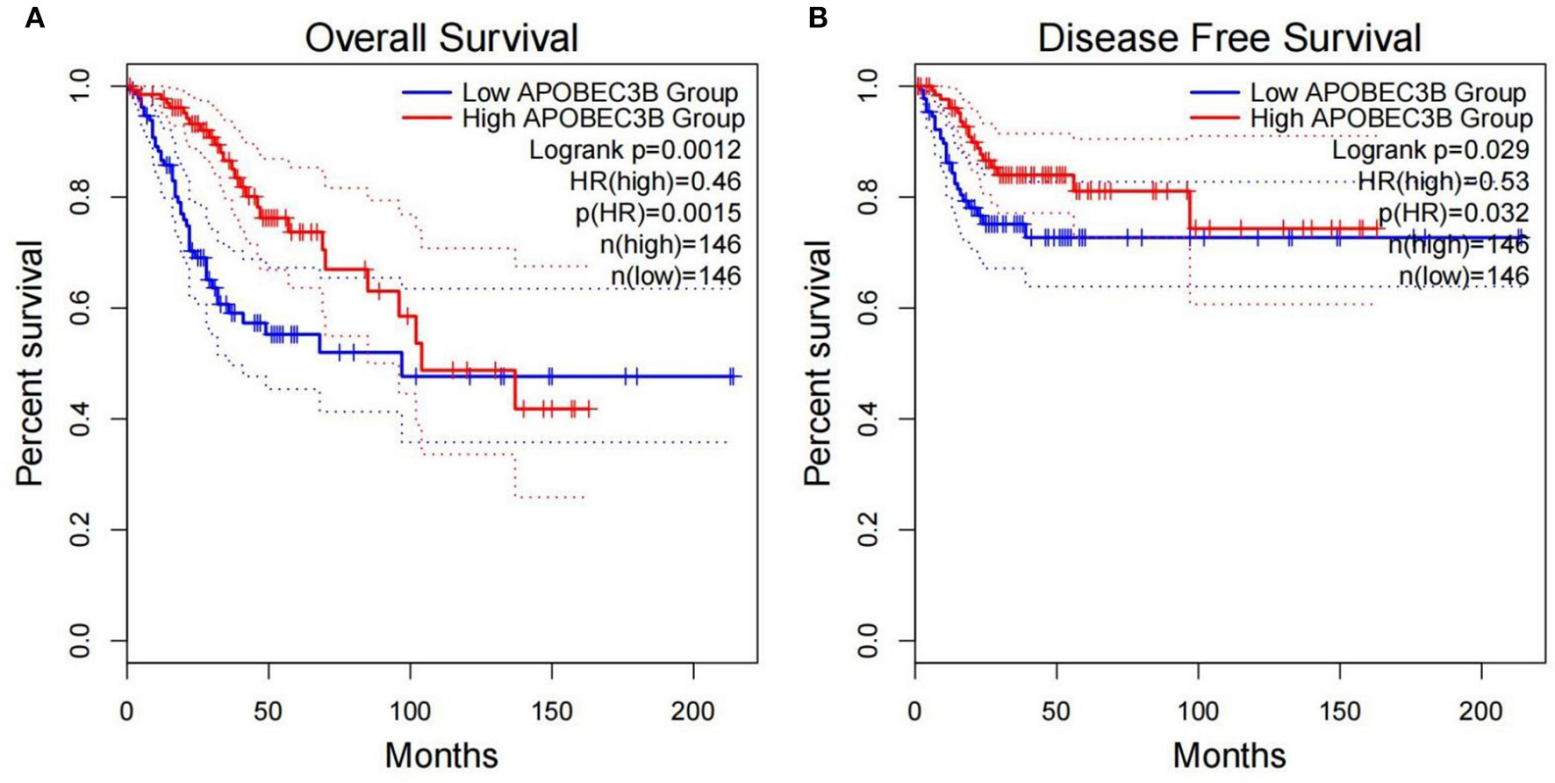
Correlation between the expression of *APOBEC3B* and overall survival (OS) and disease-free survival (DFS). **(A)** Overall survival was inferior in *APOBEC3B*-low patients; **(B)** DFS was inferior in *APOBEC3B*-low patients.

**Figure 8:**
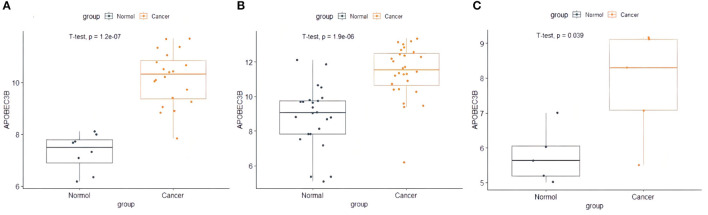
The expression of APOBEC3B in validation datasets. **(A)** The expression of APOBEC3B in GSE63678, **(B)** the expression of APOBEC3B in GSE63541, and **(C)** the expression of APOBEC3B in GES6791.

The present study performed multiple gene expression analysis of the *APOBEC* family. Among the gene expressions, it was found that the expression of *APOBEC3A* and *APOBEC3B* was significantly higher in tumor tissues than in normal tissues (Figure 6). Only the expression of *APOBEC3B* correlated with overall survival (OS) and disease-free survival (DFS) (Figure 7). Further, it was noted that only the expression of *APOBEC3B* was significantly different among the groups in the validation datasets (*p* < 0.05; Figure 8).

### Relationship Between Hub Mutant Gene and Hub Targeted Gene of the Hub Mutant Gene and Survival

**Figure 9:**
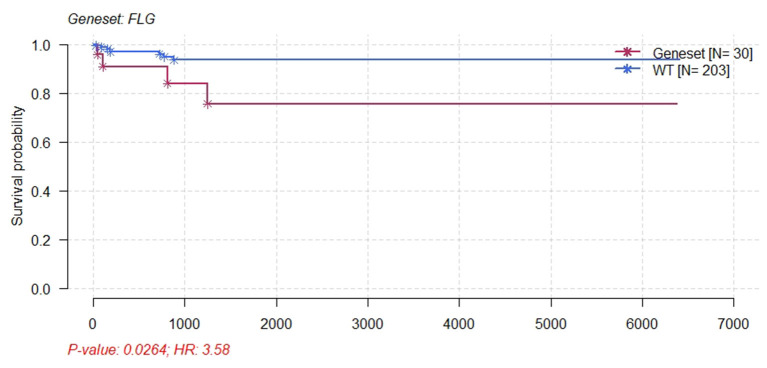
Kaplan–Meier survival plot for FLG wild-type and patients with CESC mutants. Patients with CESC and FLG mutations had significant poor survival.

**Figure 10:**
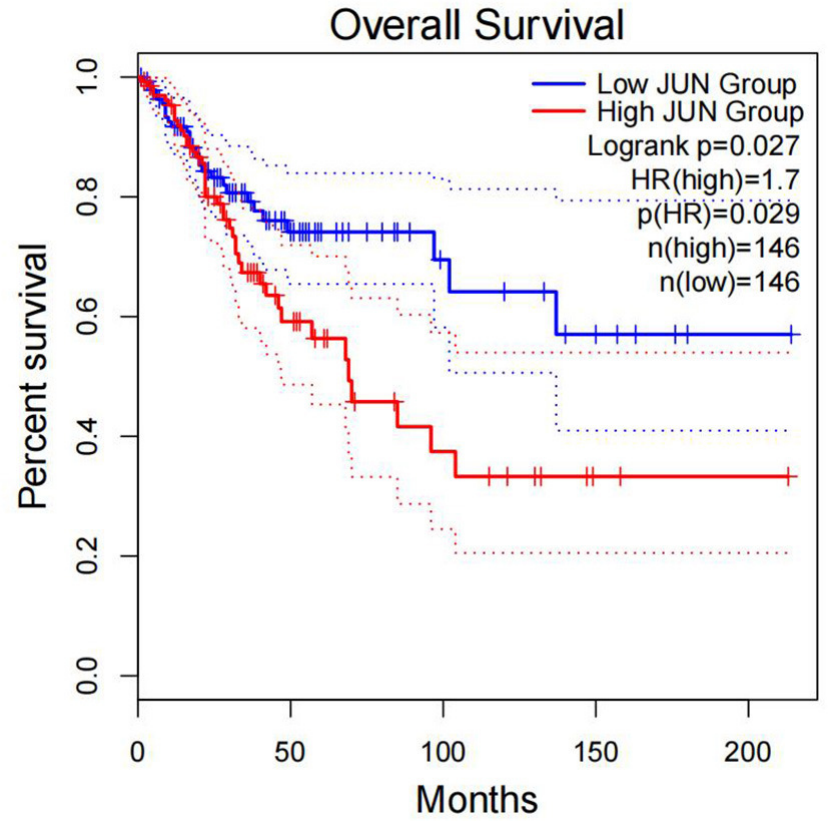
Correlation between the expression of JUN and OS. Overall survival was inferior in patients with high JUN expression.

Furthermore, survival analysis was performed in the current study using the mafsurvival function in the maftools software package. Results of the analysis found that *FLG* mutations were associated with prognosis in patients with CC (Figure 9). The patients were then classified into stages 1, 2, 3, and 4 according to FIGO staging, and it was found that the *FLG* mutations were associated with the prognosis of CC stage 1 and not with the other stages (not shown). In addition, the survival analysis of *JUN* was performed using online analysis tool-gepia2 and it was found that *JUN* was linked with prognosis of CC (Figure 10).

### Immunohistochemical Staining

**Figure 11:**
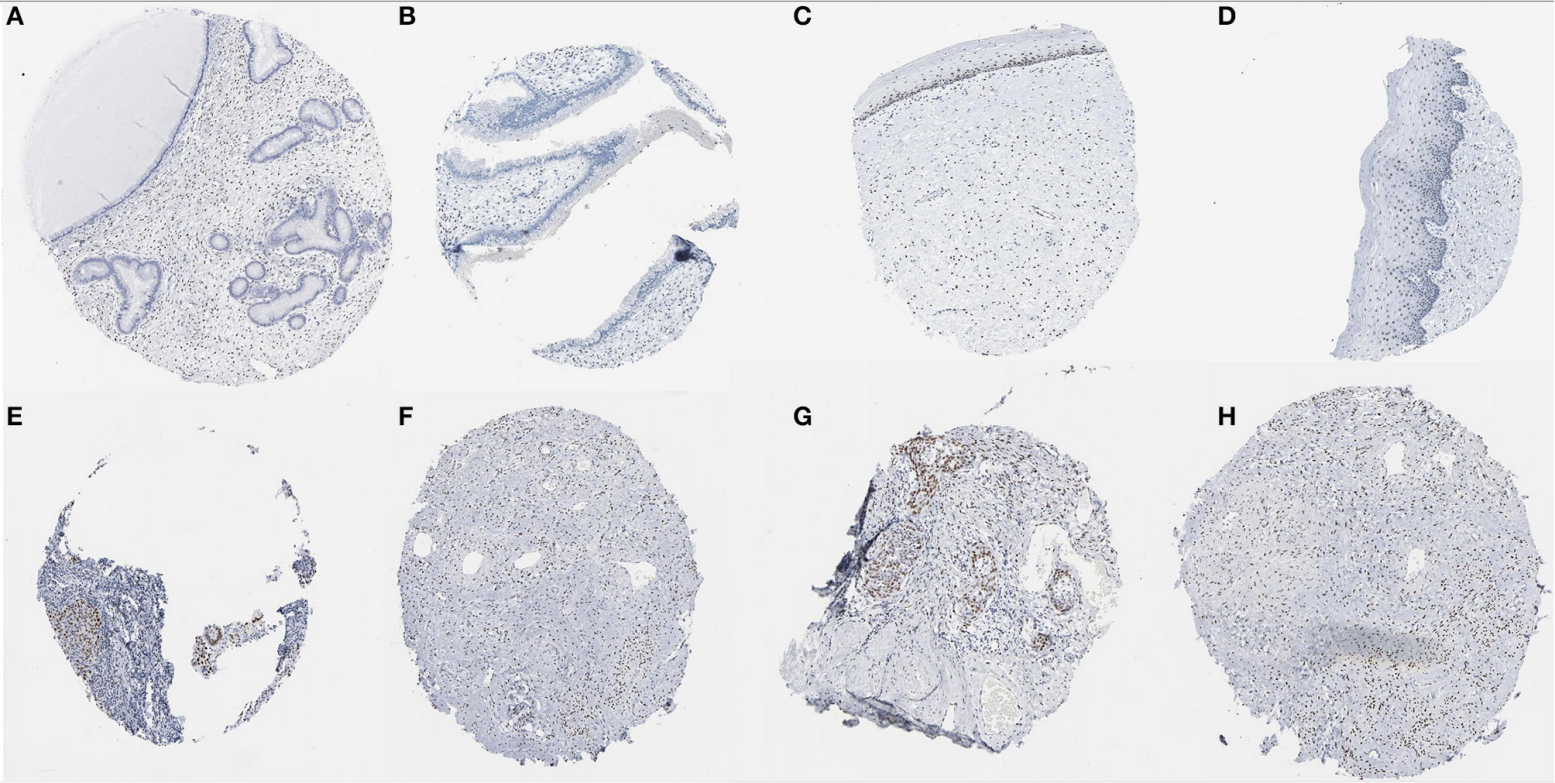
Immunohistochemical images of the JUN gene in cervical tissue. **(A–D)** Normal cervix JUN protein was not shown in normal cervical tissues; **(E–H)** JUN protein showed moderate to high levels of staining in CESC (Image available from v21.0. proteinatlas.org).

In the Human Protein Atlas website, it was found that the *JUN* protein was not shown in normal cervical tissues. However,the protein was expressed at moderate to high levels in CC tissue with two different antibodies (antibody CAB003801 and antibody CAB007780) (Figure 11).

## Discussion

The present study analyzed the masked number segment data of CECS in TCGA. It was evident that *APOBEC-*induced *FLG* gene mutations were associated with CC prognosis. Further, it was noted that the patients with *FLG* mutations had significantly lower survival rates as compared with patients with WT (*p* = 0.0246, hazard ratio [*HR*] = 3.58). In addition, online analysis through muTargets found that the expression of *JUN* was positively correlated with the mutation of *FLG* and the patients with elevated expression of *JUN* also had a poor OS rate. Results of the online analysis showed that *APOBEC3B* and *JUN* expression was higher in CC tissues than in normal tissues. Only the expression of *APOBEC3B* was correlated with OS and DFS. We validated that the expression of *APOBEC3B* was higher in CC tissues than in normal tissues across multiple independent datasets. Therefore, it is suggested that the *APOBEC-*induced mutation of *FLG* could upregulate the expression of *JUN* and then lead to a poor prognosis of patients with CC. Interestingly in this study, OS and DFS were higher in patients with high *APOBEC3B* than in those with low expression. The exact reason for this is unclear; it is possible that *APOBEC3B* plays an active role in cervical carcinogenesis, but when its expression exceeds a certain value, it regulates a certain gene or pathway to play a protective role, which needs further investigation.

The Flaggin gene is located in chromosome 1q.21.3 and its coding product is the 4,061 amino acid polypeptide chain. To the best of our knowledge, there are only two studies on *FLG* mutation of CC. First, a study conducted by Skaaby reported that *FLG* loss-of-function mutations were associated with a higher incidence of HPV-associated and precancerous cancers. Further, it was reported that the association between the *FLG* genotype and CC was statistically significant with a *HR* of 2.0 (95%; *CI*: 1.0, 4.0) (34). Elsewhere, Bager (35) reported that *FLG* mutations were not associated with CC (6.3% of cases and 7.7% of controls were carriers: OR adjusted 0.81, 95% *CI* 0.57–1.14; OR adjusted weighted 0.96, 95% *CI* 0.58–1.57). It was noted that although *FLG* mutations increased the CC mortality (*HR* 4.55, 95% *CI* 1.70–12.2), the association decreased after cancer stratification (*HR* 2.53, 95% *CI* 0.84–7.59) (35). However, the two studies were conducted on two mutant loci (c.1537C>T and c.2318-2321del) that are peculiar to the Danish population and did not address other mutant loci. Furthermore, both studies only focused on germ line mutations in the *FLG* gene.

The FLG gene is an important epidermal protein that promotes skin barrier function and its mutations remain the most easily replicated and important risk factor for atopic dermatitis (36). It has been reported that mutation of the *FLG* gene reduces or completely eliminates epidermal filament proteins and their degradation products (37). Further, the loss of *FLG* function results in a decrease in natural water, leading to an abnormal epithelial barrier (38). Oral mucosa of the defective *FLG* gene can also be prone to dryness and infection hence leading to dental caries (39). All these suggest that the *FLG* gene plays an important role in barrier function. Moreover, the filaggrin protein is notably not only expressed in the skin and oral epithelium, but also in the esophageal and cervical mucosa (34).

The *FLG* gene has been found to be significantly mutated or amplified in some types of cancer, such as colorectal carcinomas (40), differentiated thyroid cancer (41), gastric cancer (42), breast cancer (43), colorectal cancer (44), penile squamous cell carcinoma (45), glioblastoma (46), nasopharyngeal carcinoma (47), malignant melanoma (48), hepatocellular carcinoma (49), and uterine leiomyosarcoma (50). It has been reported that hairdressers are exposed to the higher levels of chemicals that result in *FLG* mutations, which may affect their risk of cancer (51). Therefore, this also suggests that *FLG* mutation takes place in response to the stimulation by external factors, thereby increasing susceptibility to cancer for people under high exposure to the factors.

Apolipoprotein B mRNA editing enzyme-catalyzed polypeptide-like (APOBEC)-induced mutations across the cancer genome are common and correlated with the levels of APOBEC mRNA. Results of TCGA-based data analysis showed that *APOBEC* mutation patterns were evident in bladder, cervical, breast, and lung as well as head and neck cancers, reaching 68% of all mutations in some samples (52). An endogenous mutational process associated with APOBEC deaminase dominates recessive dystrophic epidermolysis bullosa squamous cell carcinomas (RDEB SCC) (53).

The mutation of the *FLG* gene in the current study is a form of *APOBEC*-induced mutation. This mutation can result in the loss of function of the *FLG* gene. It has been noted that inflammation activates *APOBEC-1* and stabilizes multiple anti-apoptotic mRNAs (54). APOBEC expression plays an important role in HPV-induced CC (55). According to a study conducted by Qiong et al., *APOBEC3B* is highly expressed in CC specimens as compared with CIN III with high-risk HPV. The mechanism of APOBEC3B is that HPV-16 E6 can upregulate *APOBEC3B* by directly binding to the promoter of *APOBEC3B* in CC cells (56).

C-Jun is an important cytokine that is closely linked to the HPV life cycle (57). It has been found that the overexpression of *C-Jun* is associated with low-risk specific HPV infection in condyloma acuminatum (58). Further, the stable transfection of HPV-16 and 18 with *C-Jun* mutants reduces anchorage-independent growth (59). In addition, *JNK/c-Jun* signaling is necessary for the constitutive expression of HPV E6 and E7 that are critical for the growth and survival of CC cells (60). Therefore, *JNK/c-Jun* signaling is necessary for the survival of CC cells which suggests that *JUN* can promote HPV infection and hence lead to the development of CC.

## Conclusion

**Figure 12:**
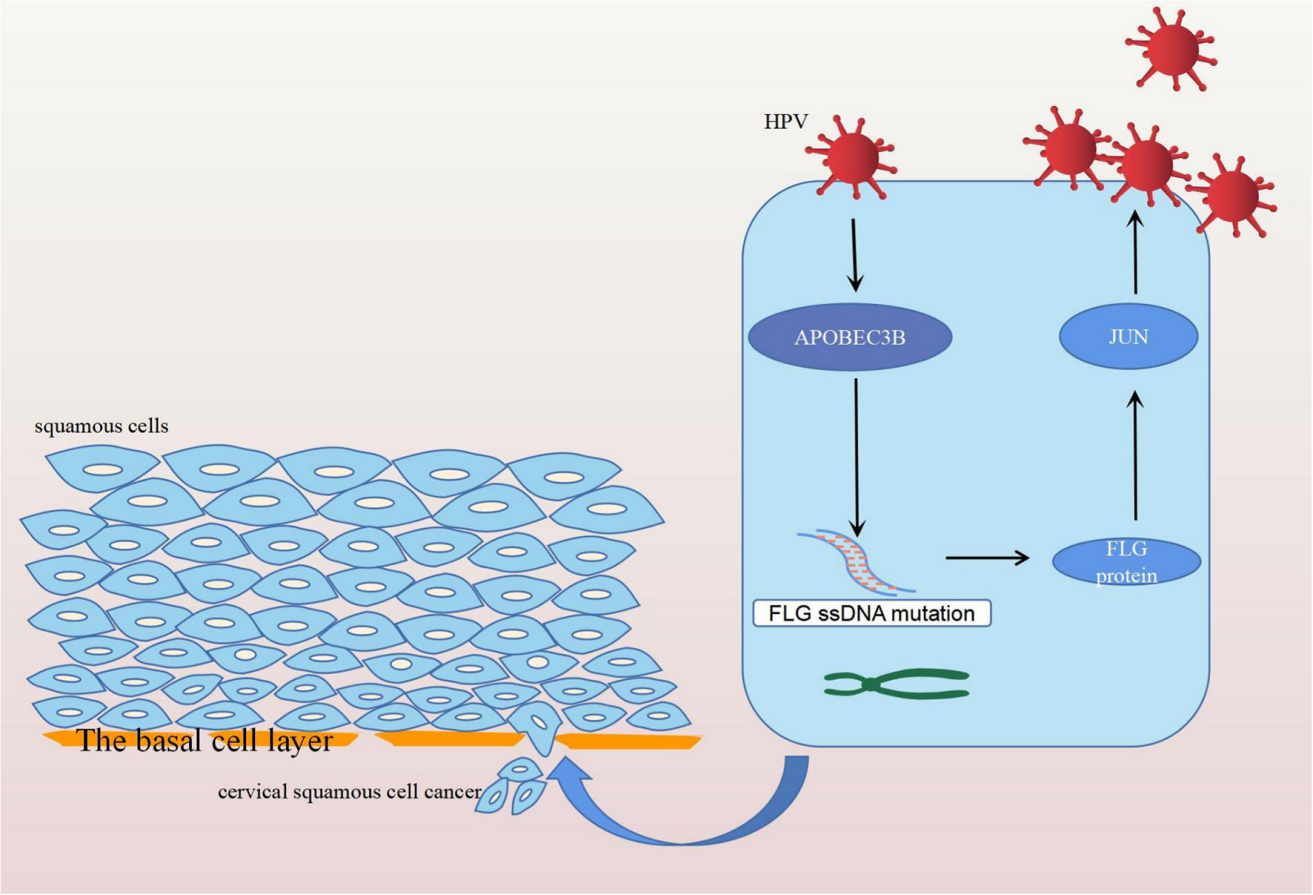
Diagram of the mechanism of action of FLG gene mutation. Human papilloma virus infection stimulates the expression of APOBEC3B, which induces the APOBEC-related mutation of FLG ssDNA. The mutated FLG protein acts on JUN, resulting in its upregulation, and ultimately leading to the development and progression of CC.

In conclusion, *FLG* gene mutation induced by *APOBEC3B* can upregulate the expression of the *JUN* gene leading to the development and progression of CC, and ultimately resulting in poor prognosis in patients with CC (Figure 12). Therefore, it is possible that the upregulation of *APOBEC3B* is due to HPV infection and this provides a new direction for the future treatment of CC.

## Data Availability Statement

The datasets presented in this study can be found in online repositories. The names of the repository/repositories and accession number(s) can be found in the article/Supplementary Material.

## Author Contributions

HC participated in the design, performed statistical analyses, and drafted the manuscript. XX conceived the study, participated in the design, and helped to draft the manuscript. LZ, JL, HZ, XW, and XF helped to draft the manuscript. All authors contributed to the article and approved the submitted version.

## Funding

This study was supported by the Fundamental Research Funds for the Central Universities of Central South University (2021zzts0391), Major Scientific and Technological Projects for collaborative prevention and control of birth defects in Hunan Province (2019SK1010), the National Natural Science Foundation of China (Grant Number 81771558), and Natural Science Foundation of Hunan Province (Grant Number 2020JJ4814).

## Conflict of Interest

The authors declare that the research was conducted in the absence of any commercial or financial relationships that could be construed as a potential conflict of interest.

## Publisher's Note

All claims expressed in this article are solely those of the authors and do not necessarily represent those of their affiliated organizations, or those of the publisher, the editors and the reviewers. Any product that may be evaluated in this article, or claim that may be made by its manufacturer, is not guaranteed or endorsed by the publisher.
