# Supplementary material for: Bioinformatic Analyzes of the Association Between Upregulated Expression of JUN Gene via APOBEC-Induced FLG Gene Mutation and Prognosis of Cervical Cancer
Source: Front Med (Lausanne). 2022 Apr 18;9:815450. doi: 10.3389/fmed.2022.815450 (PMC9058067; doi:10.3389/fmed.2022.815450)
Supplement: Supplementary file 1 [file Data_Sheet_1.ZIP › Enrichment_GO/ColorByCluster.pdf]

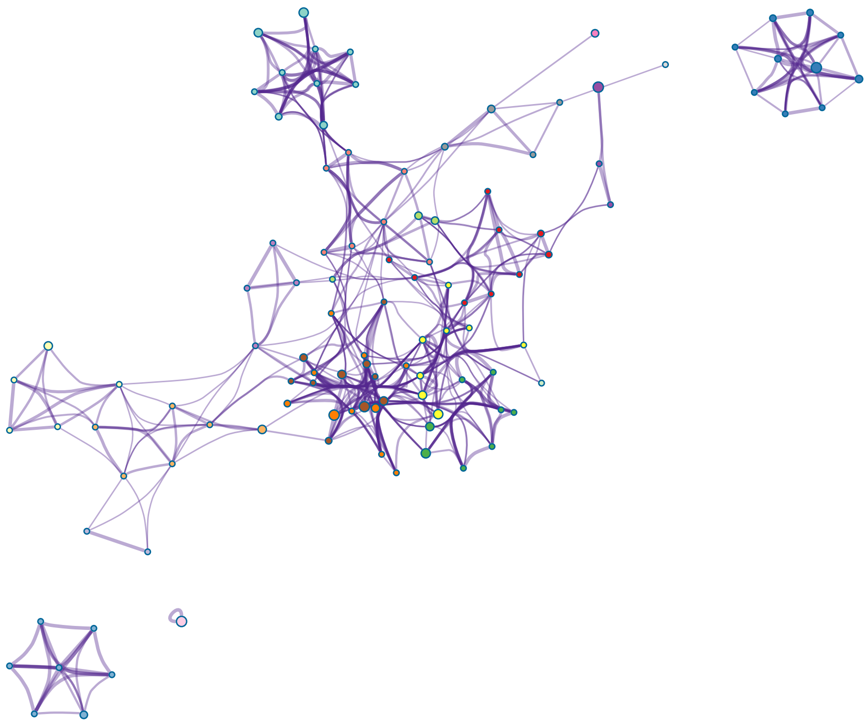

- digestive system process
- O-glycan processing
- regulation of body fluid levels
- multicellular organismal homeostasis
- urogenital system development
- inner ear development
- regulation of hemopoiesis
- organic hydroxy compound transport
- cellular hormone metabolic process
- positive regulation of cellular component biogenesis
- viral process
- mucosal immune response
- oligodendrocyte development
- purine ribonucleotide catabolic process
- toll-like receptor 4 signaling pathway
- response to metal ion
- response to bacterium
- cellular aldehyde metabolic process
- positive regulation of mononuclear cell migration
- fat cell differentiation

created by
http://metascape.org
