# Supplementary figures and images for: Bioinformatic Analyzes of the Association Between Upregulated Expression of JUN Gene via APOBEC-Induced FLG Gene Mutation and Prognosis of Cervical Cancer
Source: Front Med (Lausanne). 2022 Apr 18;9:815450. doi: 10.3389/fmed.2022.815450 (PMC9058067; doi:10.3389/fmed.2022.815450)

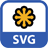

Supplement: Supplementary file 1 [file Data_Sheet_1.ZIP › icon/SVG48.png]

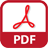

Supplement: Supplementary file 1 [file Data_Sheet_1.ZIP › icon/PDF48.png]

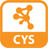

Supplement: Supplementary file 1 [file Data_Sheet_1.ZIP › icon/CYS48.png]

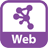

Supplement: Supplementary file 1 [file Data_Sheet_1.ZIP › icon/WEB_CYS48.png]

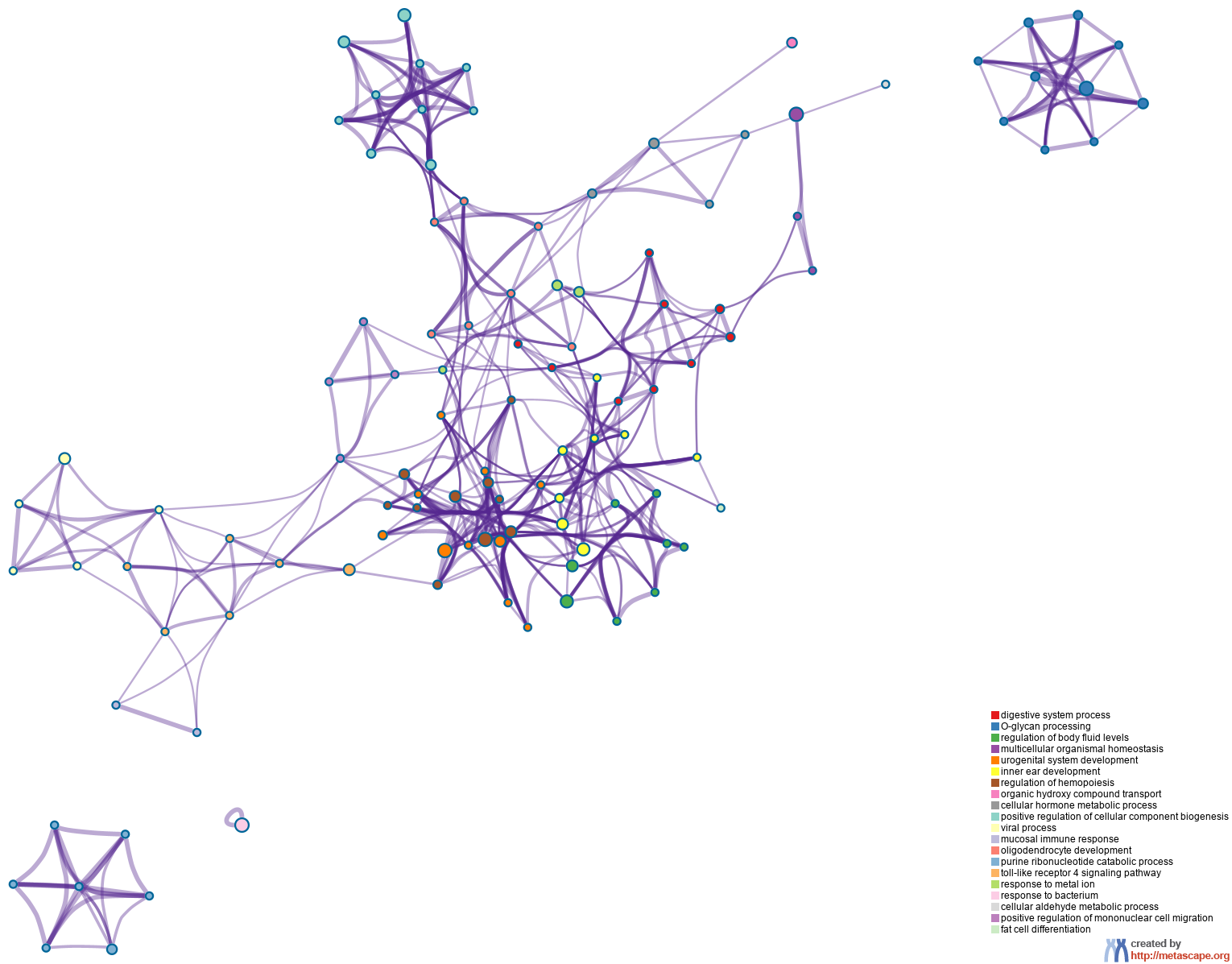

Supplement: Supplementary file 1 [file Data_Sheet_1.ZIP › Enrichment_GO/ColorByCluster.png]

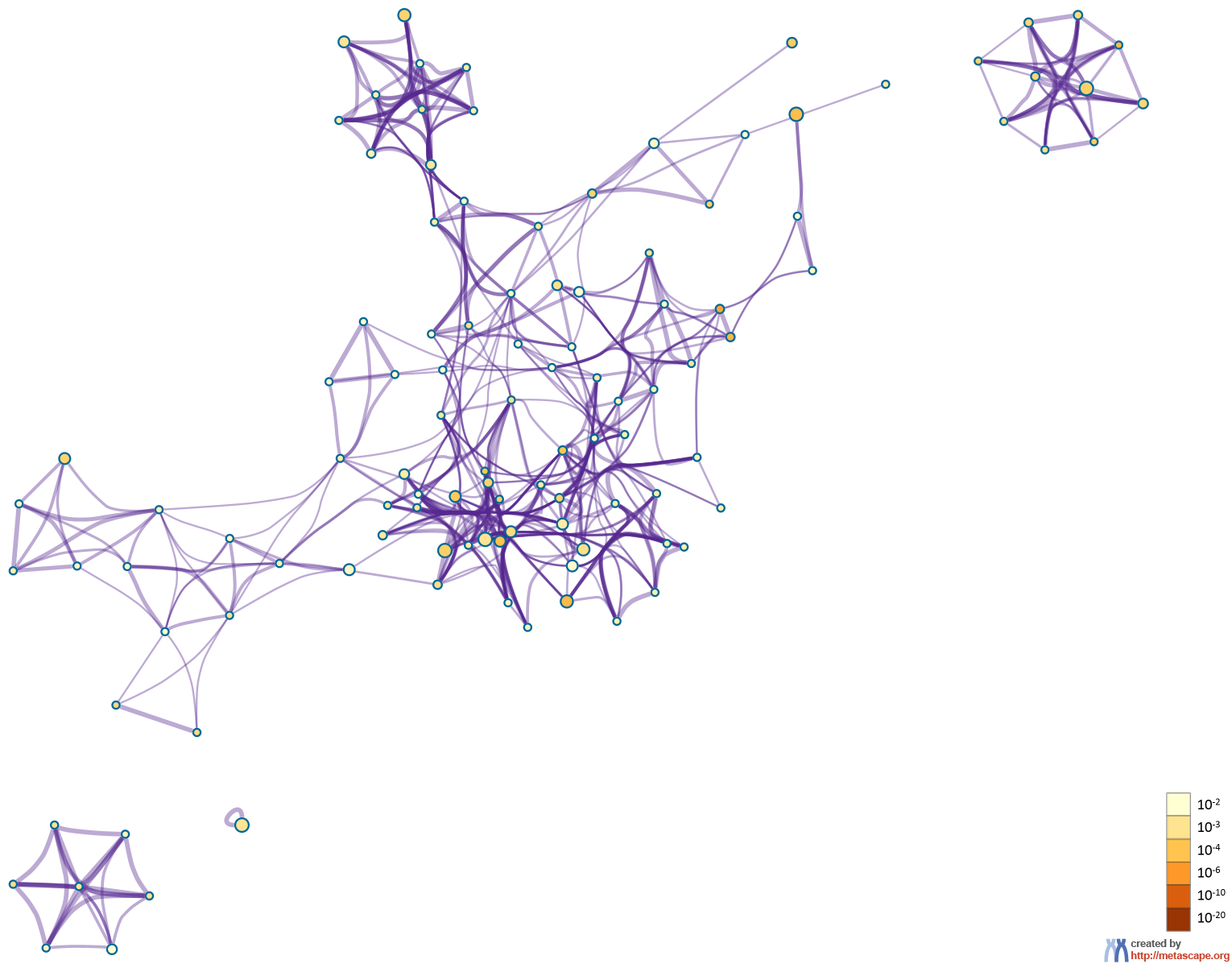

Supplement: Supplementary file 1 [file Data_Sheet_1.ZIP › Enrichment_GO/ColorByPValue.png]

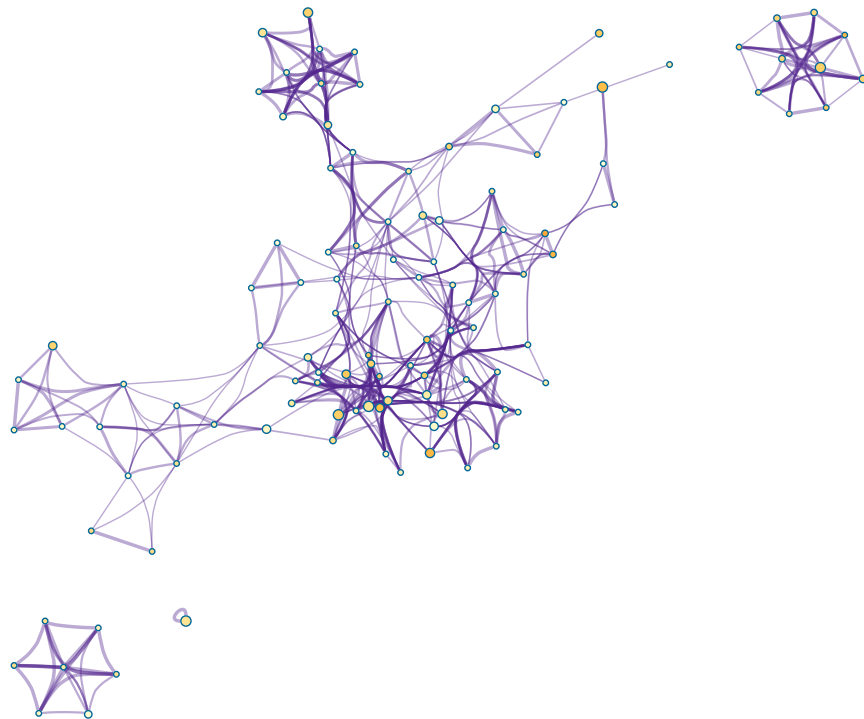

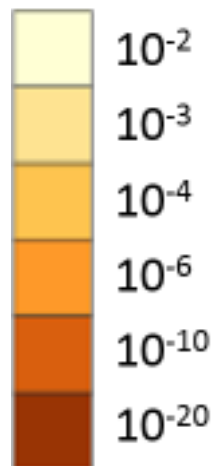

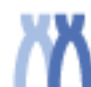 created by  
<http://metascape.org>

Supplement: Supplementary file 1 [file Data_Sheet_1.ZIP › Enrichment_GO/ColorByPValue.pdf]

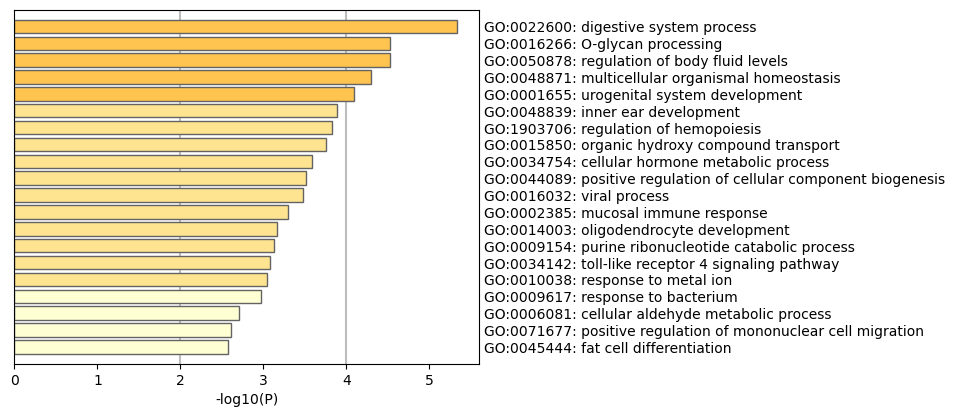

Supplement: Supplementary file 1 [file Data_Sheet_1.ZIP › Enrichment_heatmap/HeatmapSelectedGO.png]

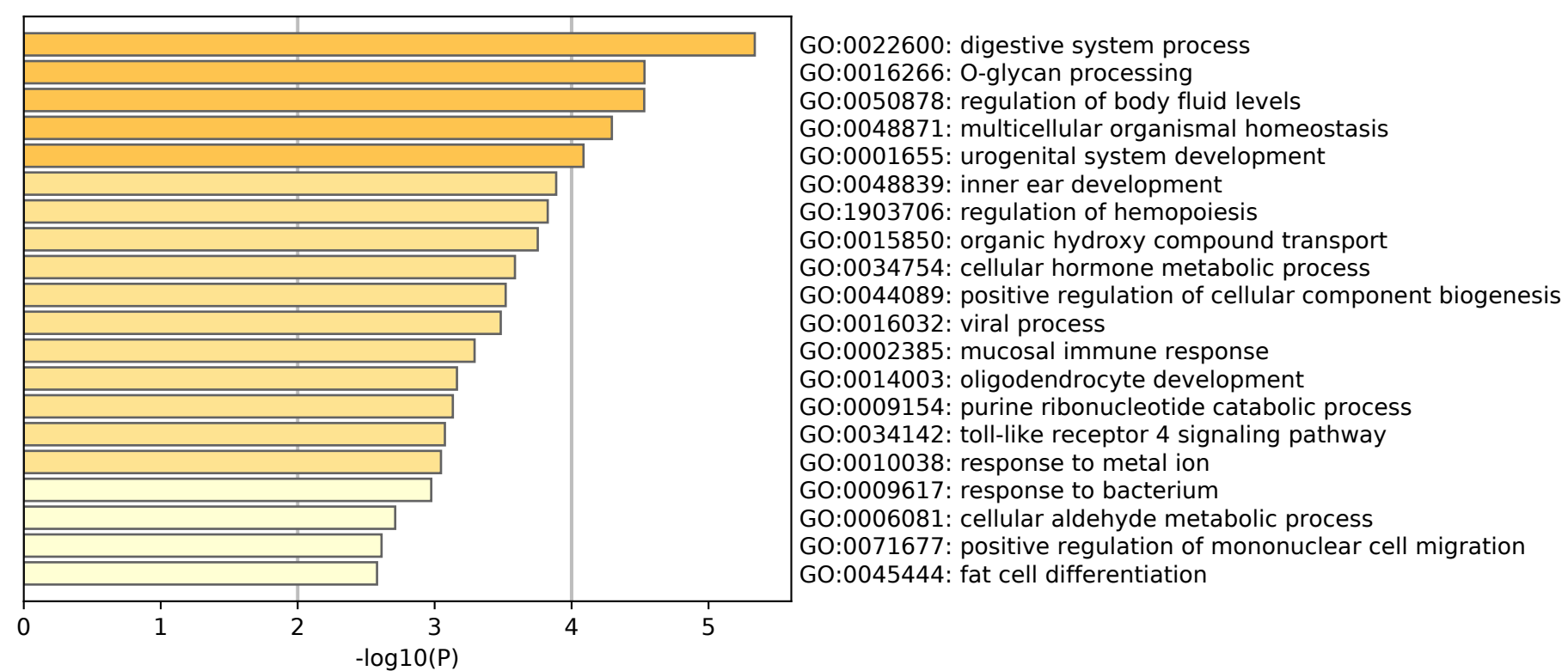

Supplement: Supplementary file 1 [file Data_Sheet_1.ZIP › Enrichment_heatmap/HeatmapSelectedGO.pdf]

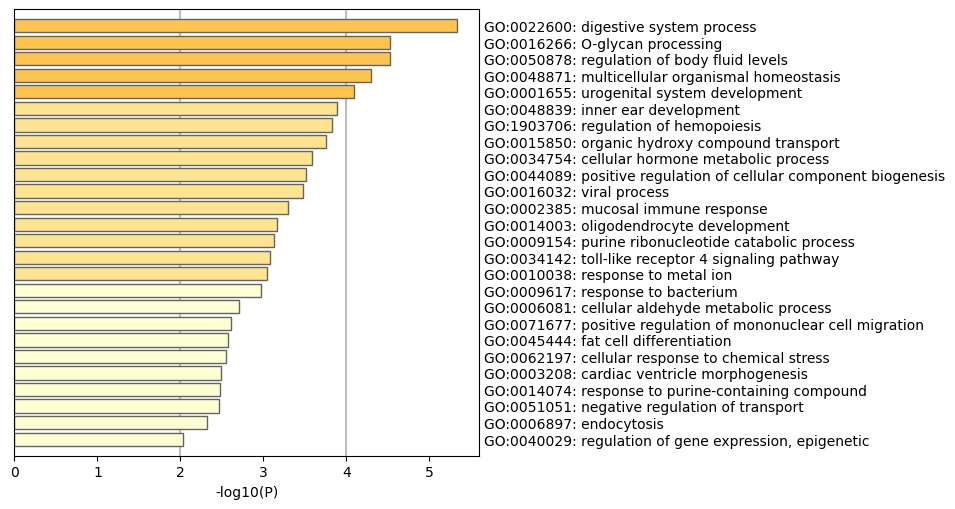

Supplement: Supplementary file 1 [file Data_Sheet_1.ZIP › Enrichment_heatmap/HeatmapSelectedGOTop100.png]

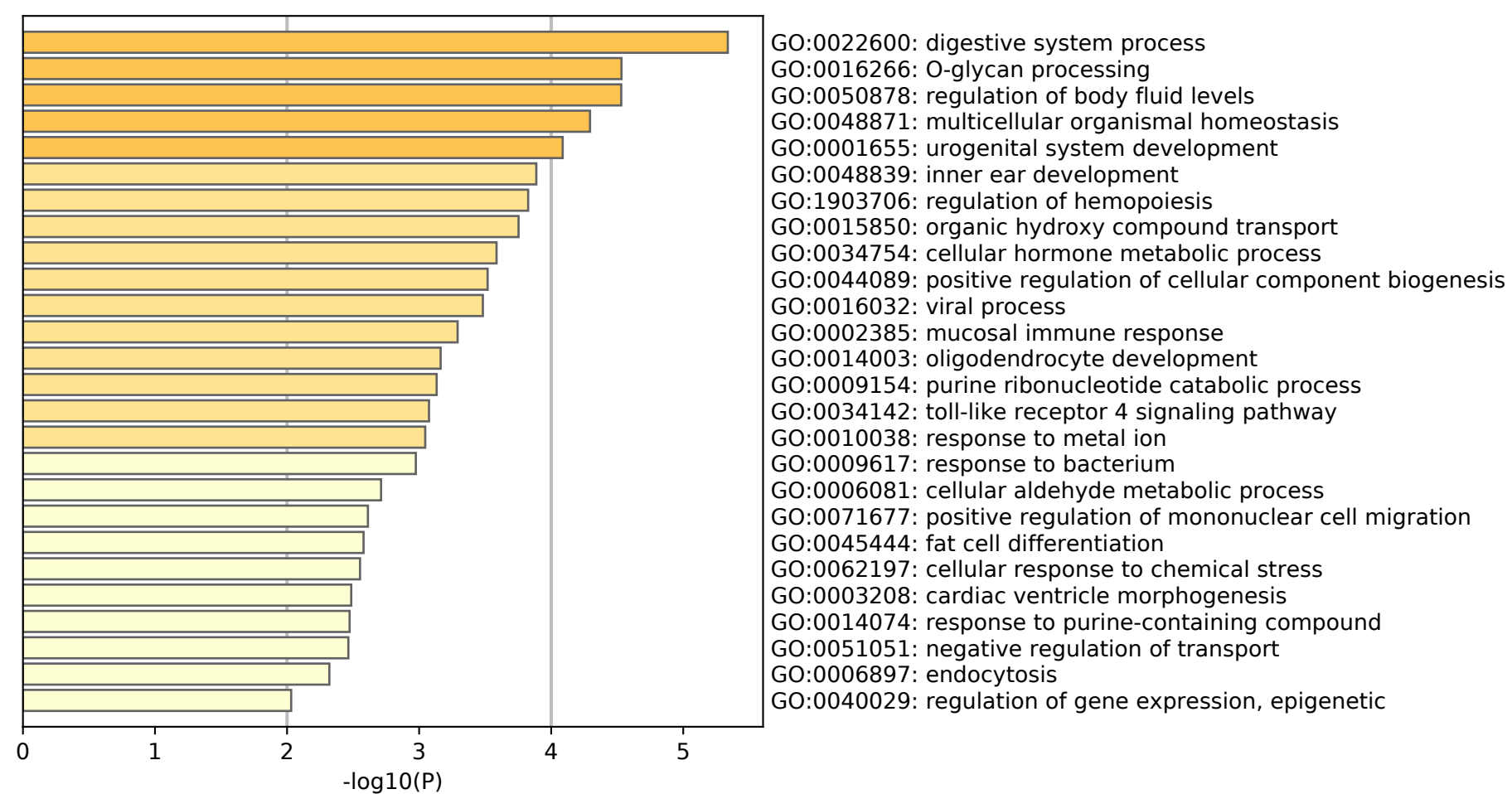

Supplement: Supplementary file 1 [file Data_Sheet_1.ZIP › Enrichment_heatmap/HeatmapSelectedGOTop100.pdf]

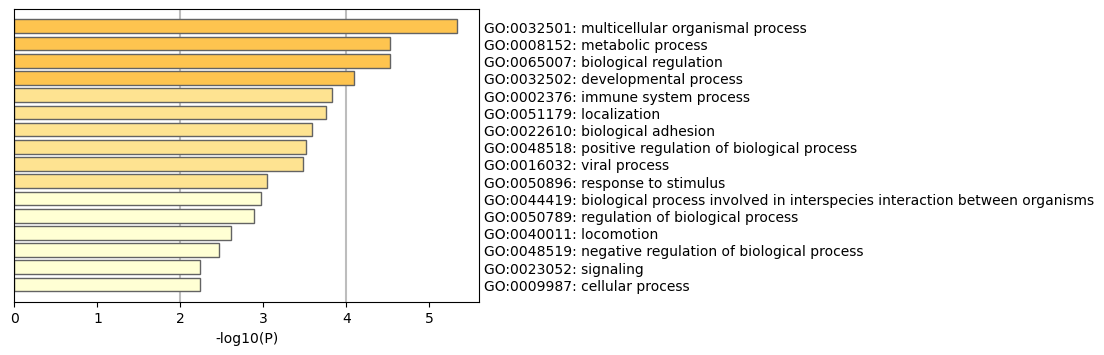

Supplement: Supplementary file 1 [file Data_Sheet_1.ZIP › Enrichment_heatmap/HeatmapSelectedGOParent.png]

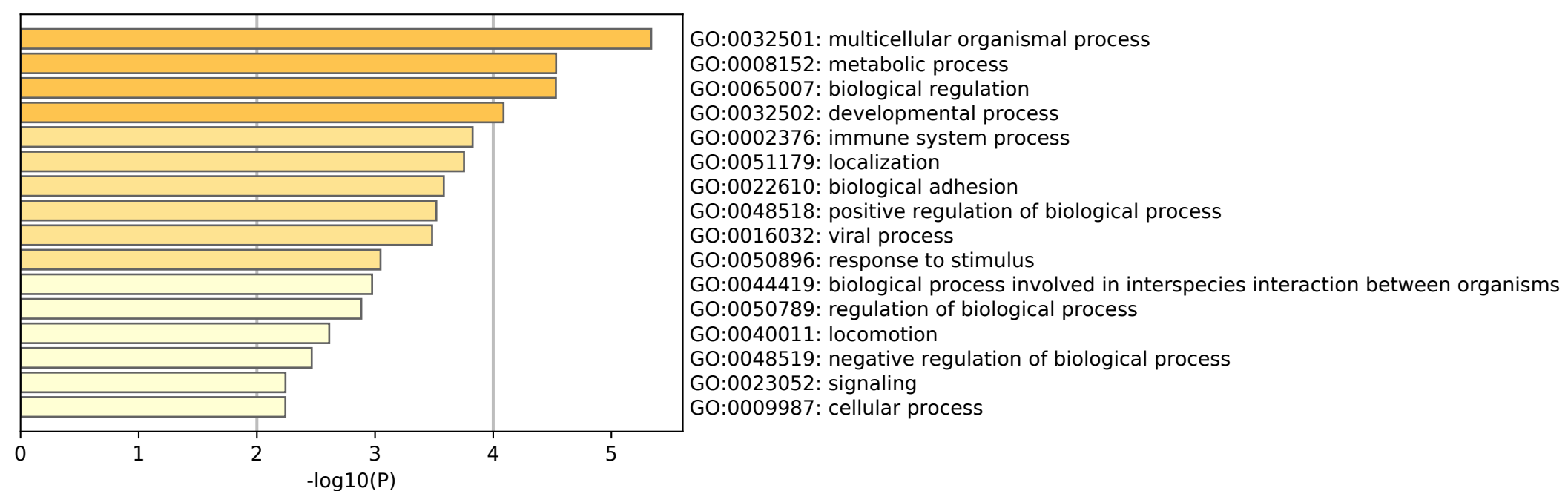

Supplement: Supplementary file 1 [file Data_Sheet_1.ZIP › Enrichment_heatmap/HeatmapSelectedGOParent.pdf]

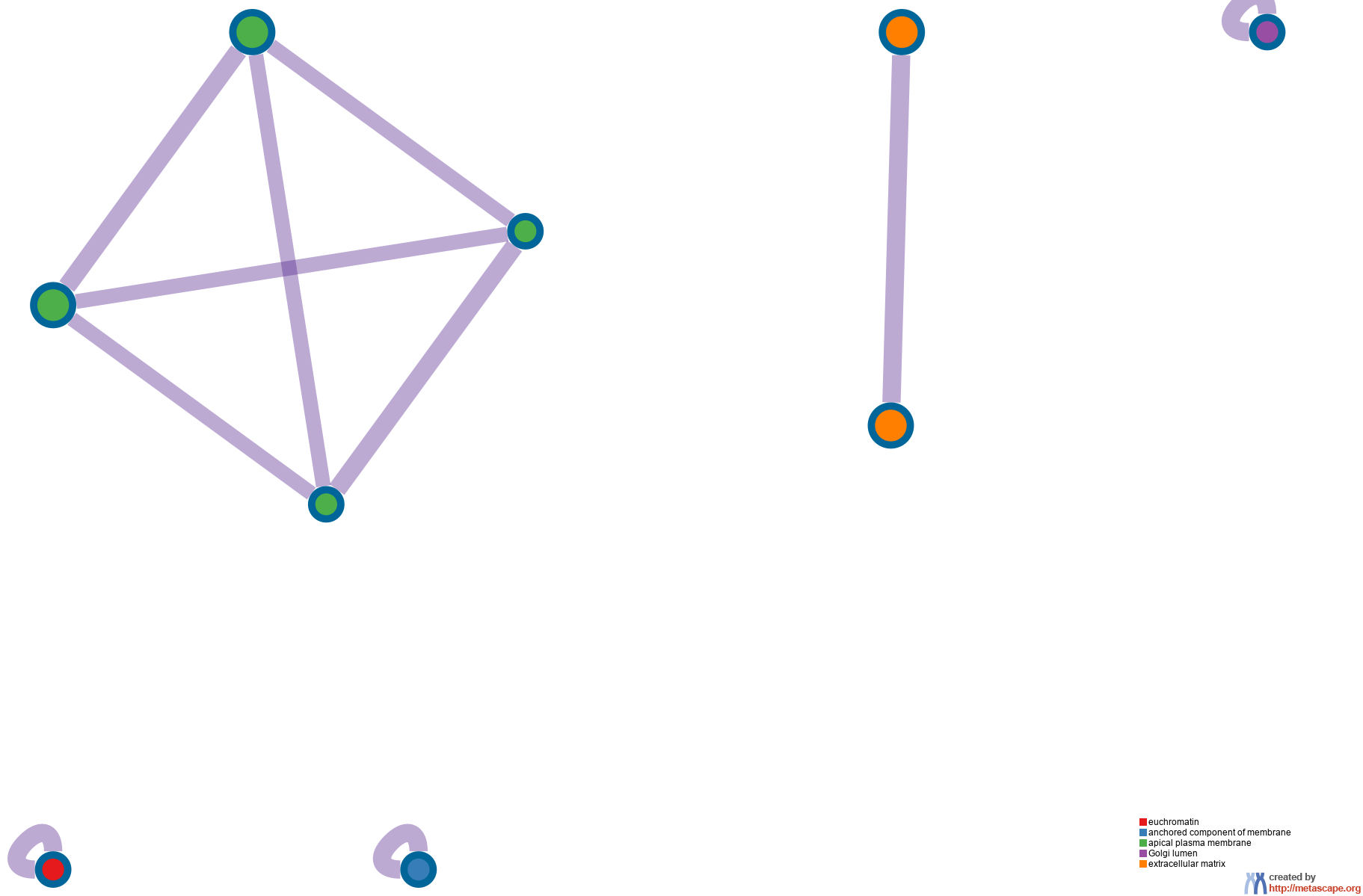

Supplement: Supplementary file 2 [file Data_Sheet_2.ZIP › Enrichment_GO/ColorByCluster.png]

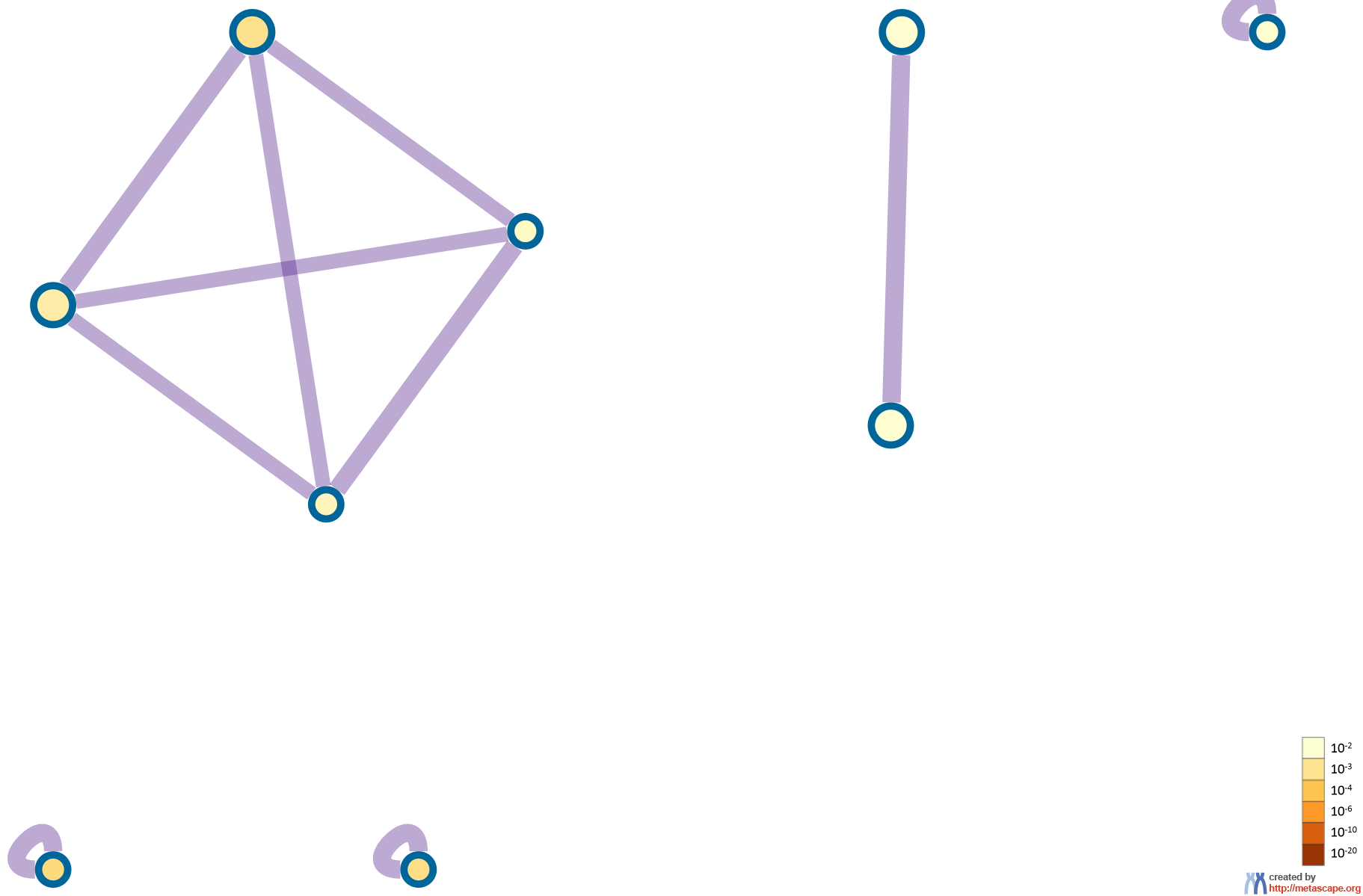

Supplement: Supplementary file 2 [file Data_Sheet_2.ZIP › Enrichment_GO/ColorByPValue.png]

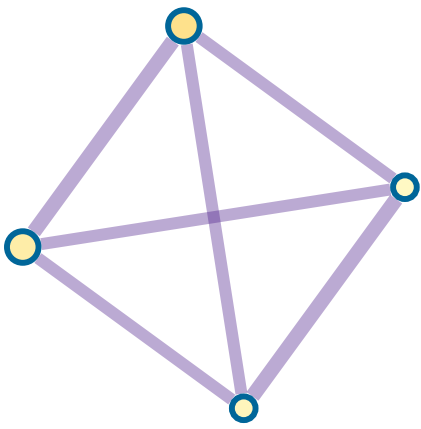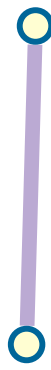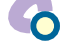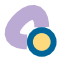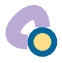

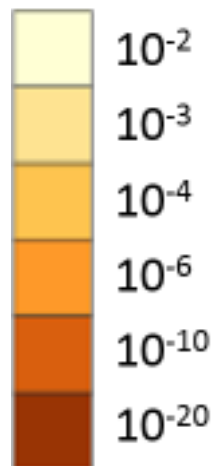

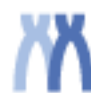 created by  
<http://metascape.org>

Supplement: Supplementary file 2 [file Data_Sheet_2.ZIP › Enrichment_GO/ColorByPValue.pdf]

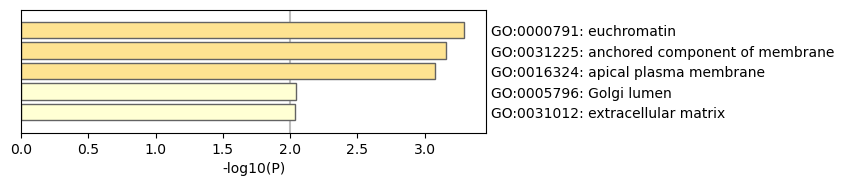

Supplement: Supplementary file 2 [file Data_Sheet_2.ZIP › Enrichment_heatmap/HeatmapSelectedGO.png]

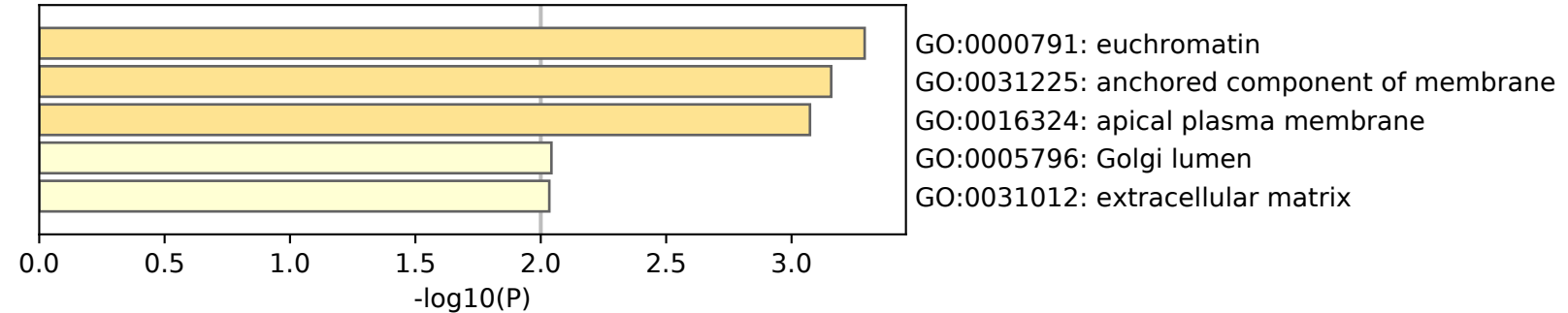

Supplement: Supplementary file 2 [file Data_Sheet_2.ZIP › Enrichment_heatmap/HeatmapSelectedGO.pdf]

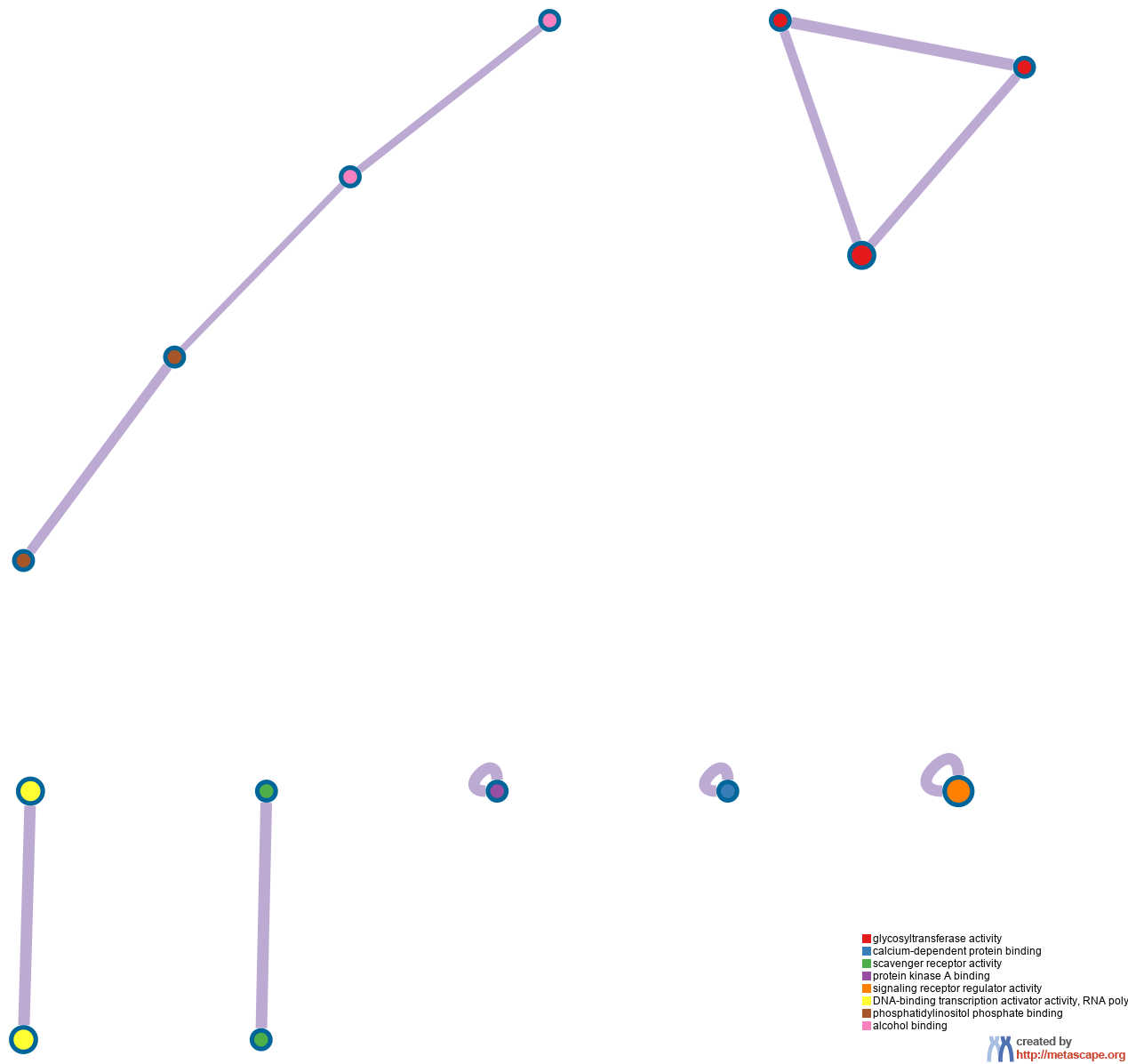

Supplement: Supplementary file 3 [file Data_Sheet_3.ZIP › Enrichment_GO/ColorByCluster.png]

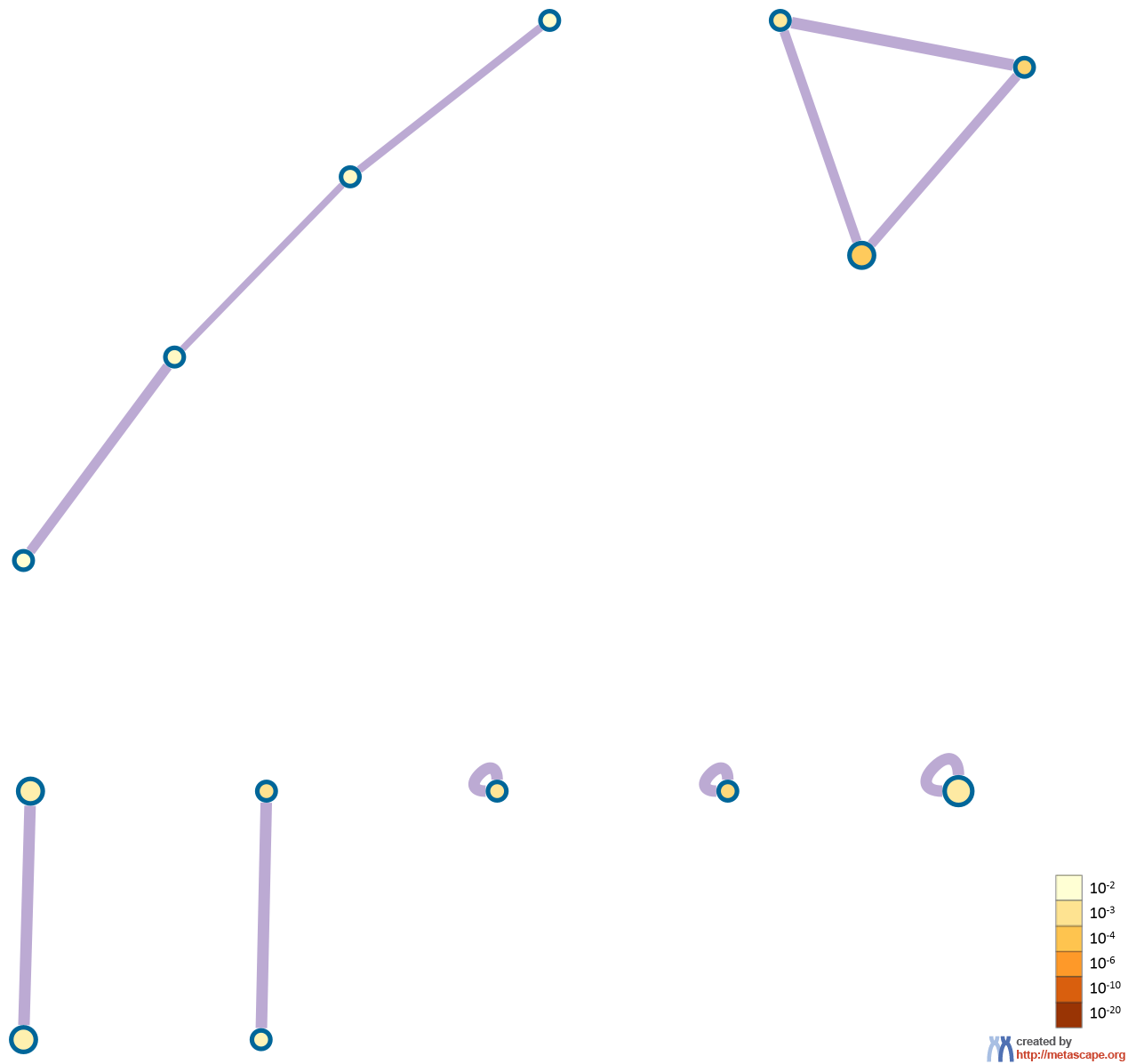

Supplement: Supplementary file 3 [file Data_Sheet_3.ZIP › Enrichment_GO/ColorByPValue.png]

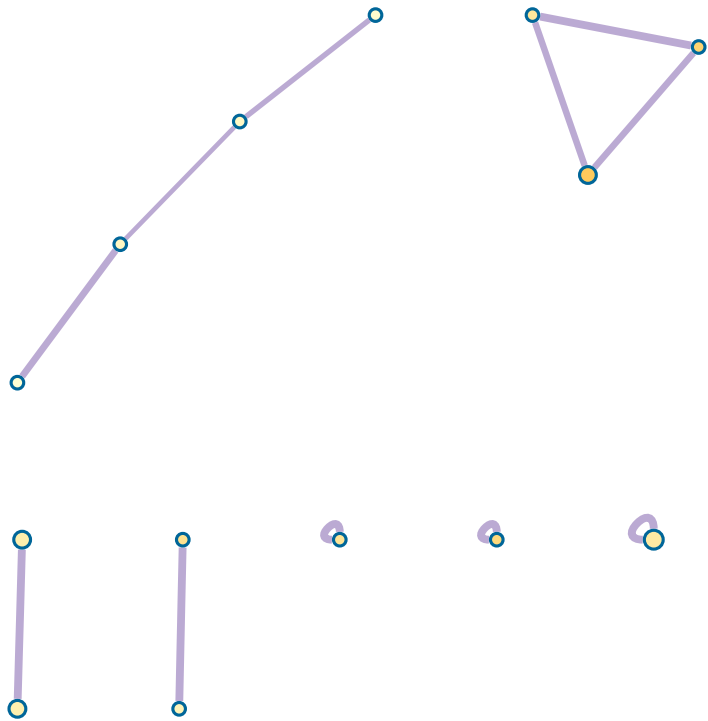

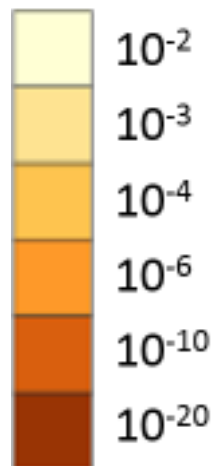

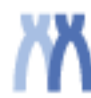 created by  
<http://metascape.org>

Supplement: Supplementary file 3 [file Data_Sheet_3.ZIP › Enrichment_GO/ColorByPValue.pdf]

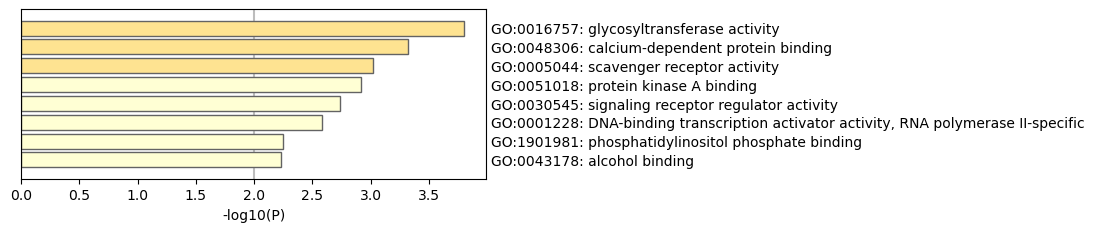

Supplement: Supplementary file 3 [file Data_Sheet_3.ZIP › Enrichment_heatmap/HeatmapSelectedGO.png]

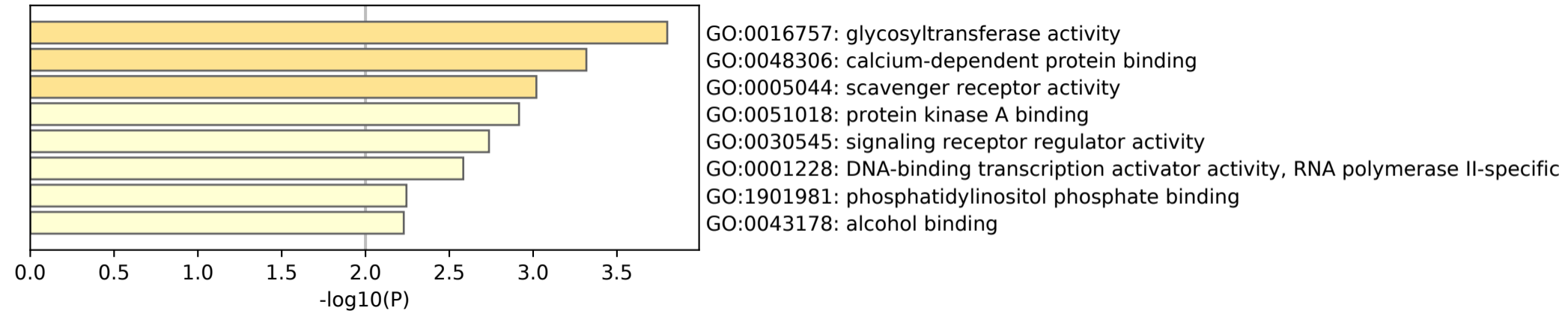

Supplement: Supplementary file 3 [file Data_Sheet_3.ZIP › Enrichment_heatmap/HeatmapSelectedGO.pdf]

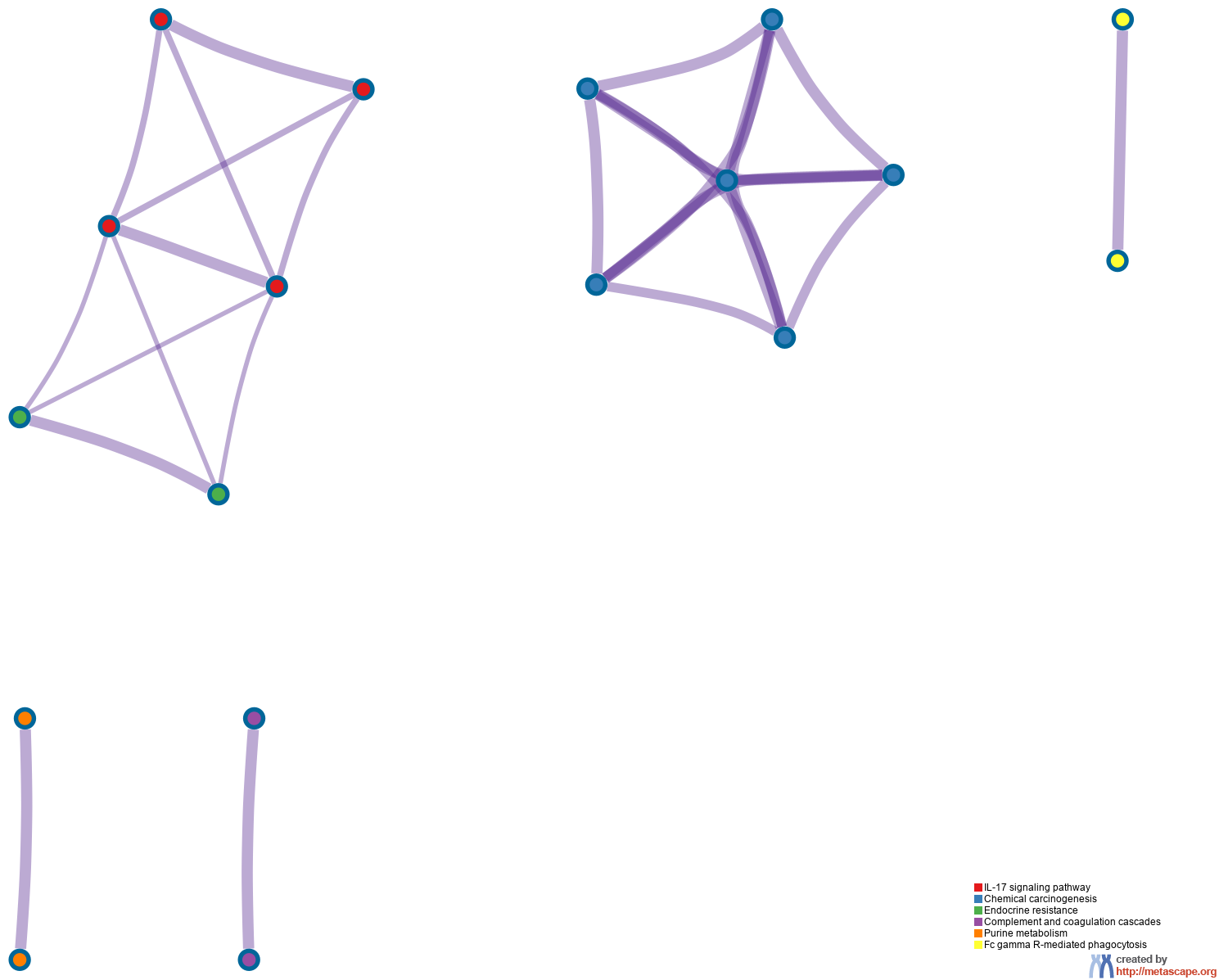

Supplement: Supplementary file 4 [file Data_Sheet_4.ZIP › Enrichment_GO/ColorByCluster.png]

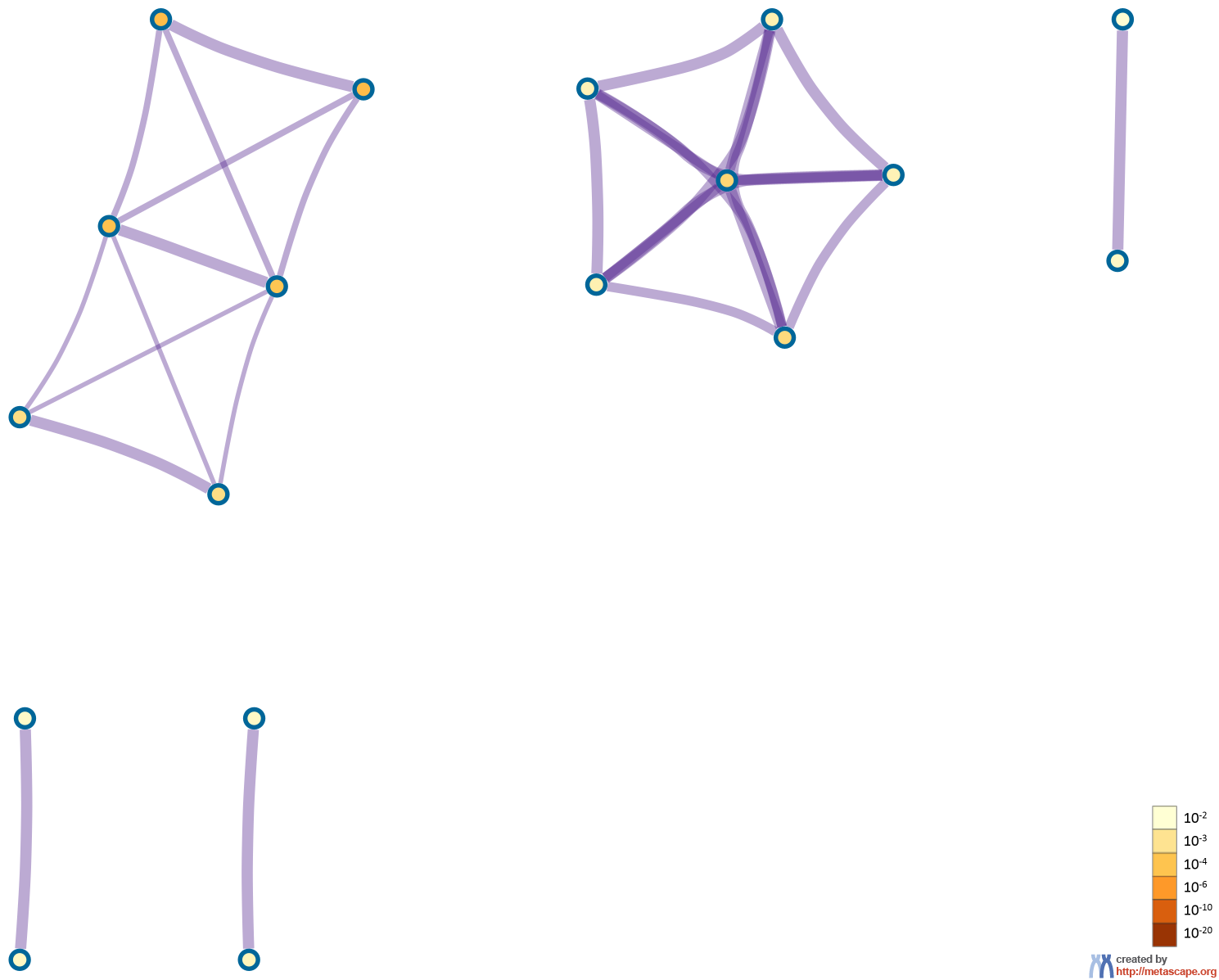

Supplement: Supplementary file 4 [file Data_Sheet_4.ZIP › Enrichment_GO/ColorByPValue.png]

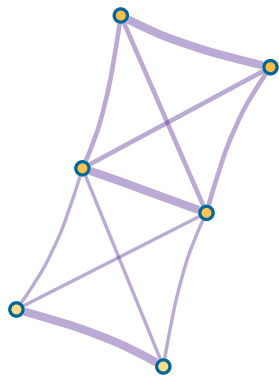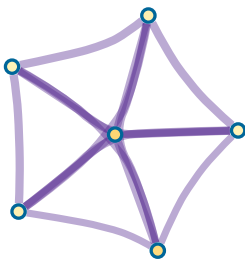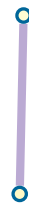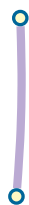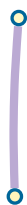

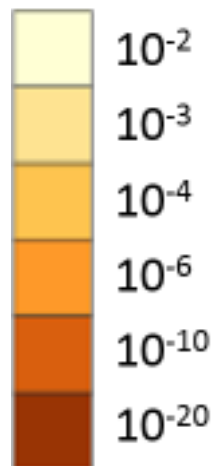

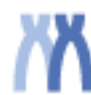 created by  
<http://metascape.org>

Supplement: Supplementary file 4 [file Data_Sheet_4.ZIP › Enrichment_GO/ColorByPValue.pdf]

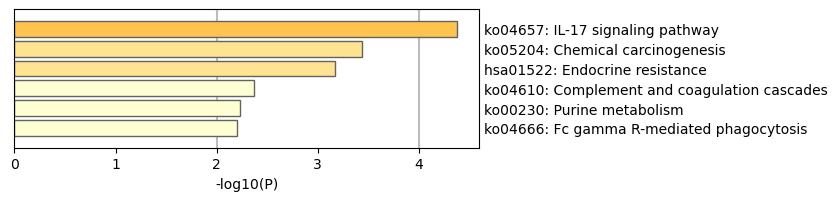

Supplement: Supplementary file 4 [file Data_Sheet_4.ZIP › Enrichment_heatmap/HeatmapSelectedGO.png]

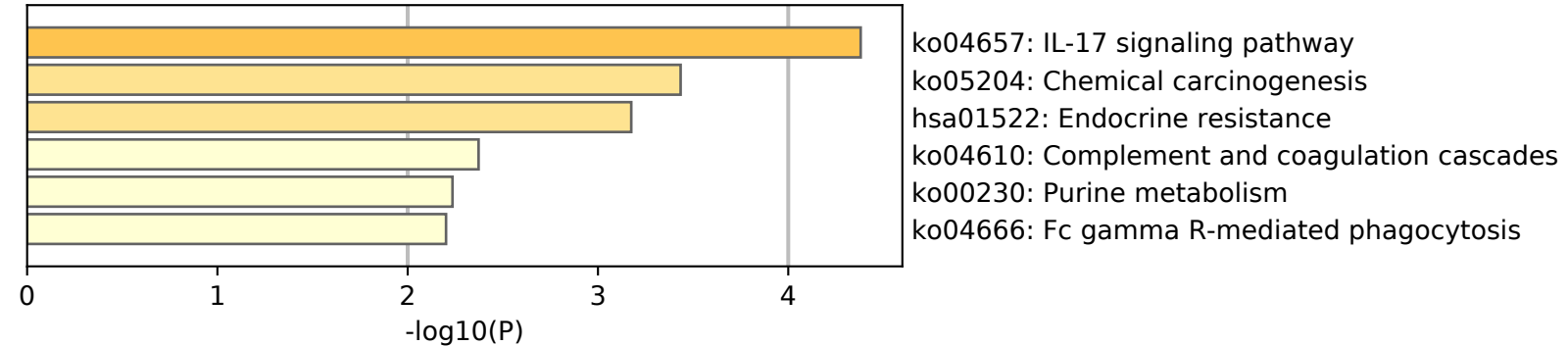

Supplement: Supplementary file 4 [file Data_Sheet_4.ZIP › Enrichment_heatmap/HeatmapSelectedGO.pdf]
