# Supplementary material for: Bioinformatic Analyzes of the Association Between Upregulated Expression of JUN Gene via APOBEC-Induced FLG Gene Mutation and Prognosis of Cervical Cancer
Source: Front Med (Lausanne). 2022 Apr 18;9:815450. doi: 10.3389/fmed.2022.815450 (PMC9058067; doi:10.3389/fmed.2022.815450)
Supplement: Supplementary file 2 [file Data_Sheet_2.ZIP › Enrichment_GO/ColorByCluster.pdf]

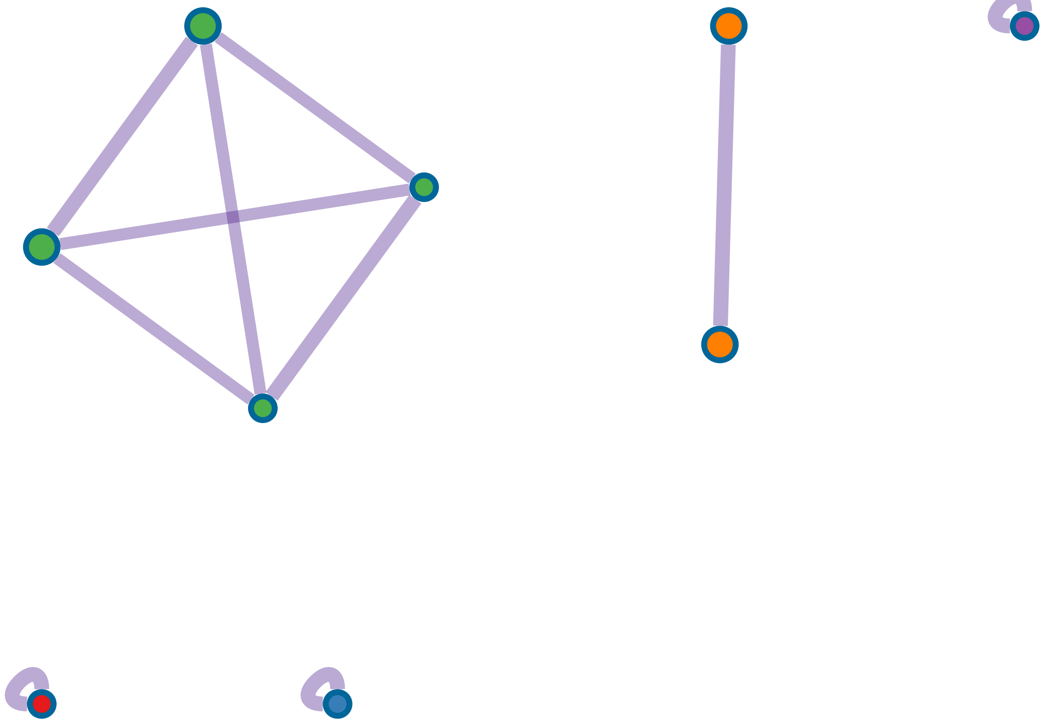

euchromatin
anchored component of membrane
apical plasma membrane
Golgi lumen
extracellular matrix

created by
http://metascape.org
