# Supplementary material for: Bioinformatic Analyzes of the Association Between Upregulated Expression of JUN Gene via APOBEC-Induced FLG Gene Mutation and Prognosis of Cervical Cancer
Source: Front Med (Lausanne). 2022 Apr 18;9:815450. doi: 10.3389/fmed.2022.815450 (PMC9058067; doi:10.3389/fmed.2022.815450)
Supplement: Supplementary file 3 [file Data_Sheet_3.ZIP › AnalysisReport.pptx]

## Slide 1
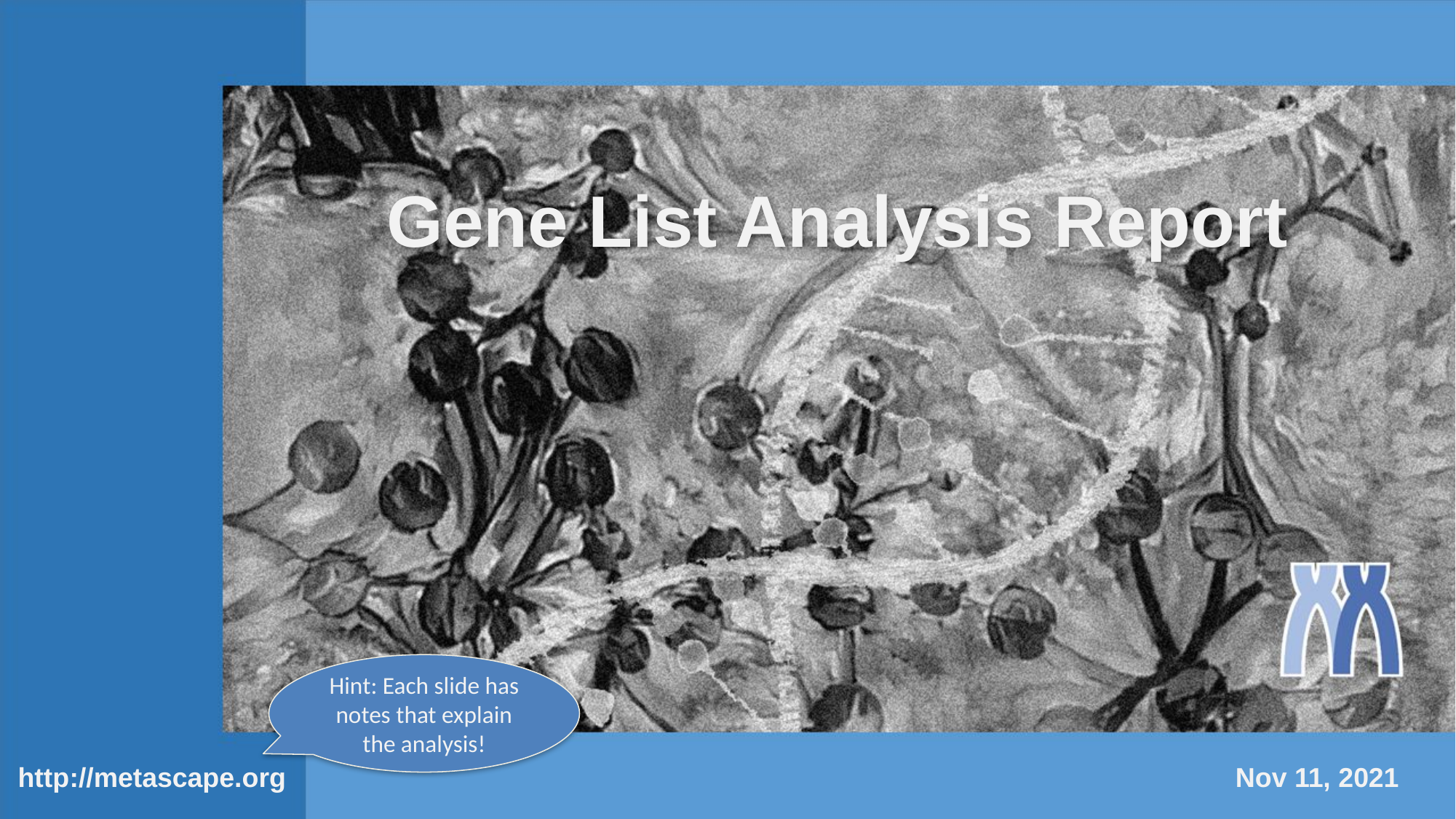

# Gene List Analysis Report
Hint: Each slide has notes that explain the analysis!
http://metascape.org
Nov 11, 2021

## Slide 2
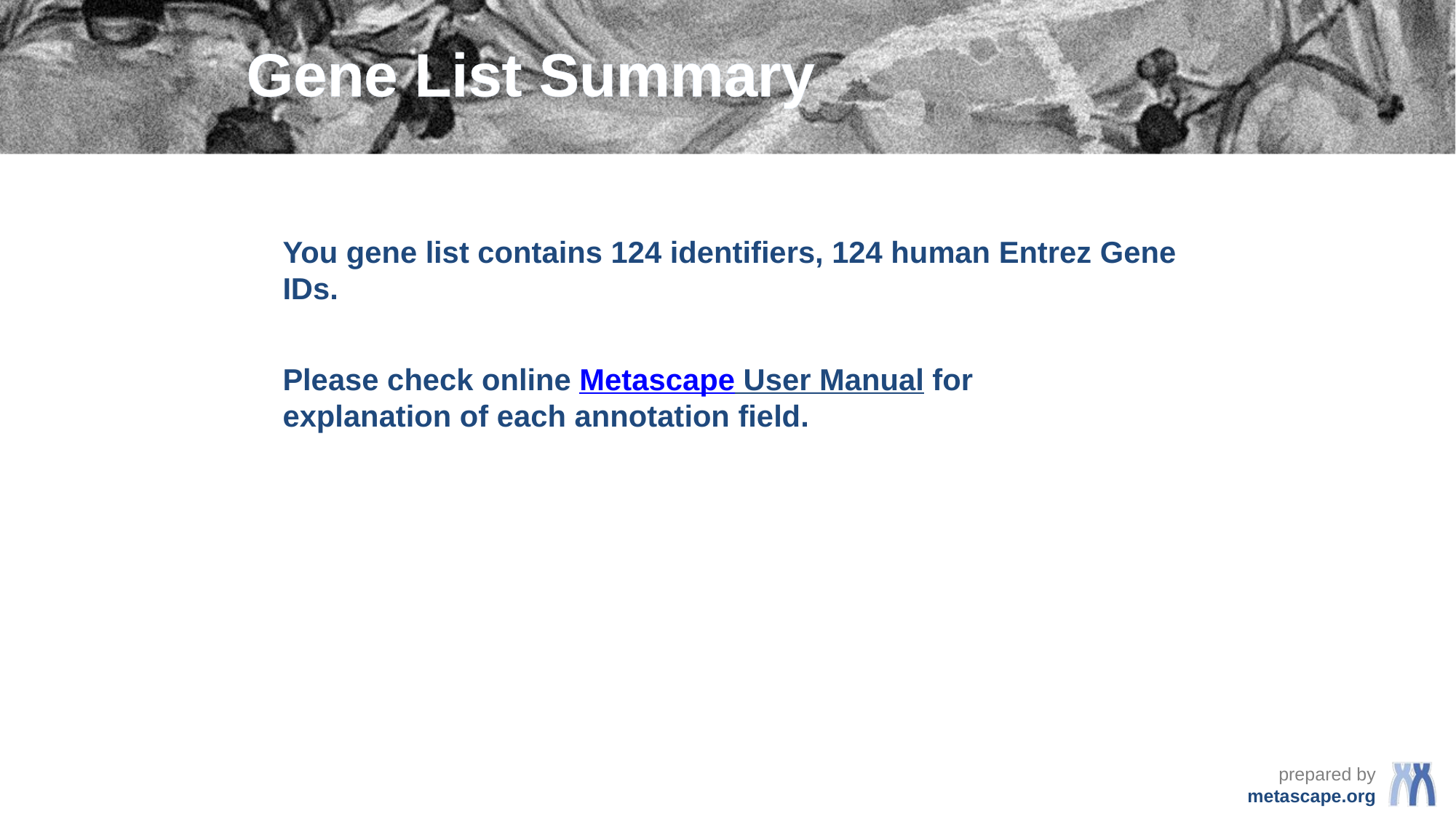

# Gene List Summary
You gene list contains 124 identifiers, 124 human Entrez Gene IDs.
Please check online Metascape User Manual for explanation of each annotation field.

## Slide 3
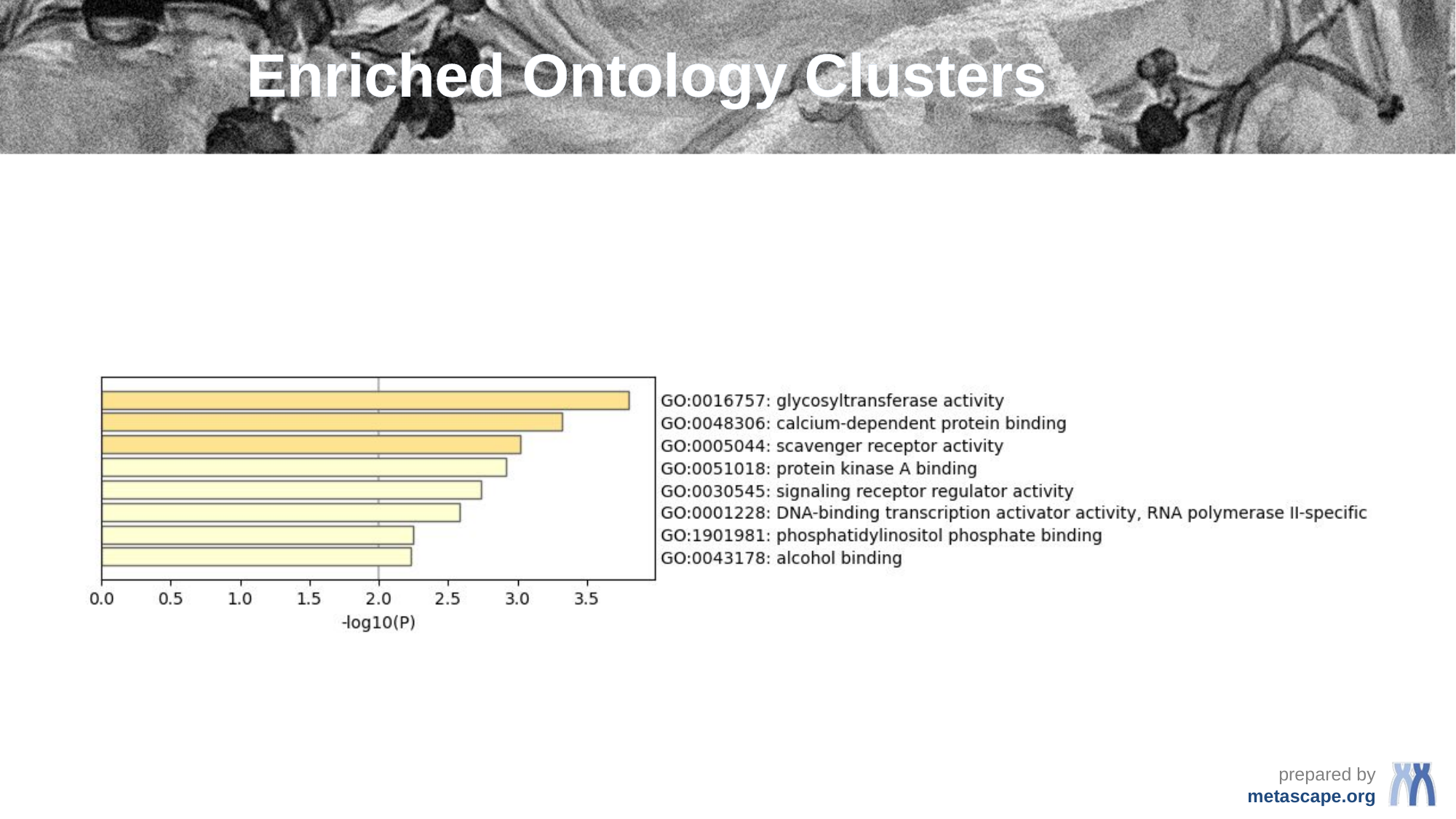

# Enriched Ontology Clusters

## Slide 4
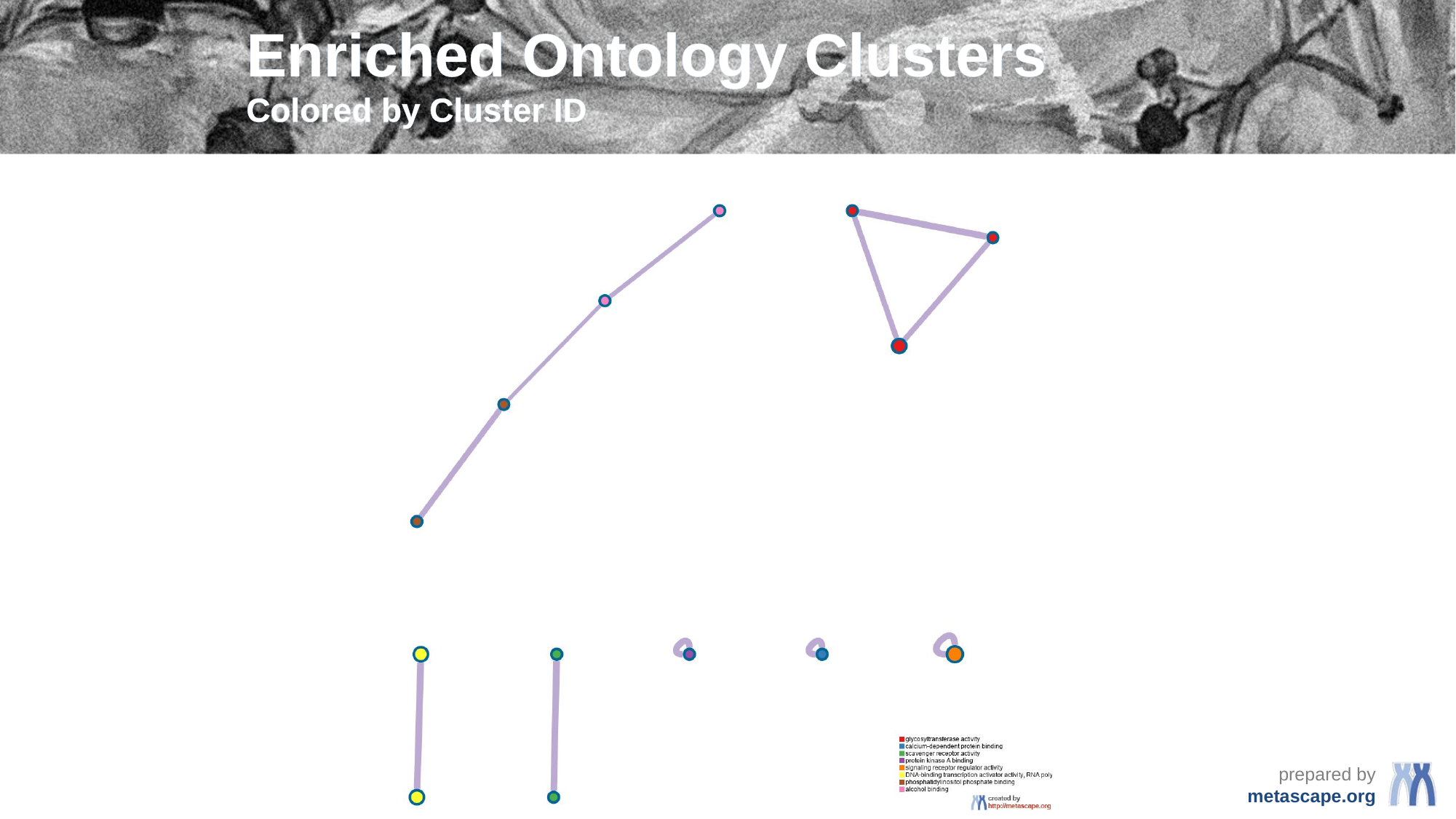

# Enriched Ontology Clusters Colored by Cluster ID

## Slide 5
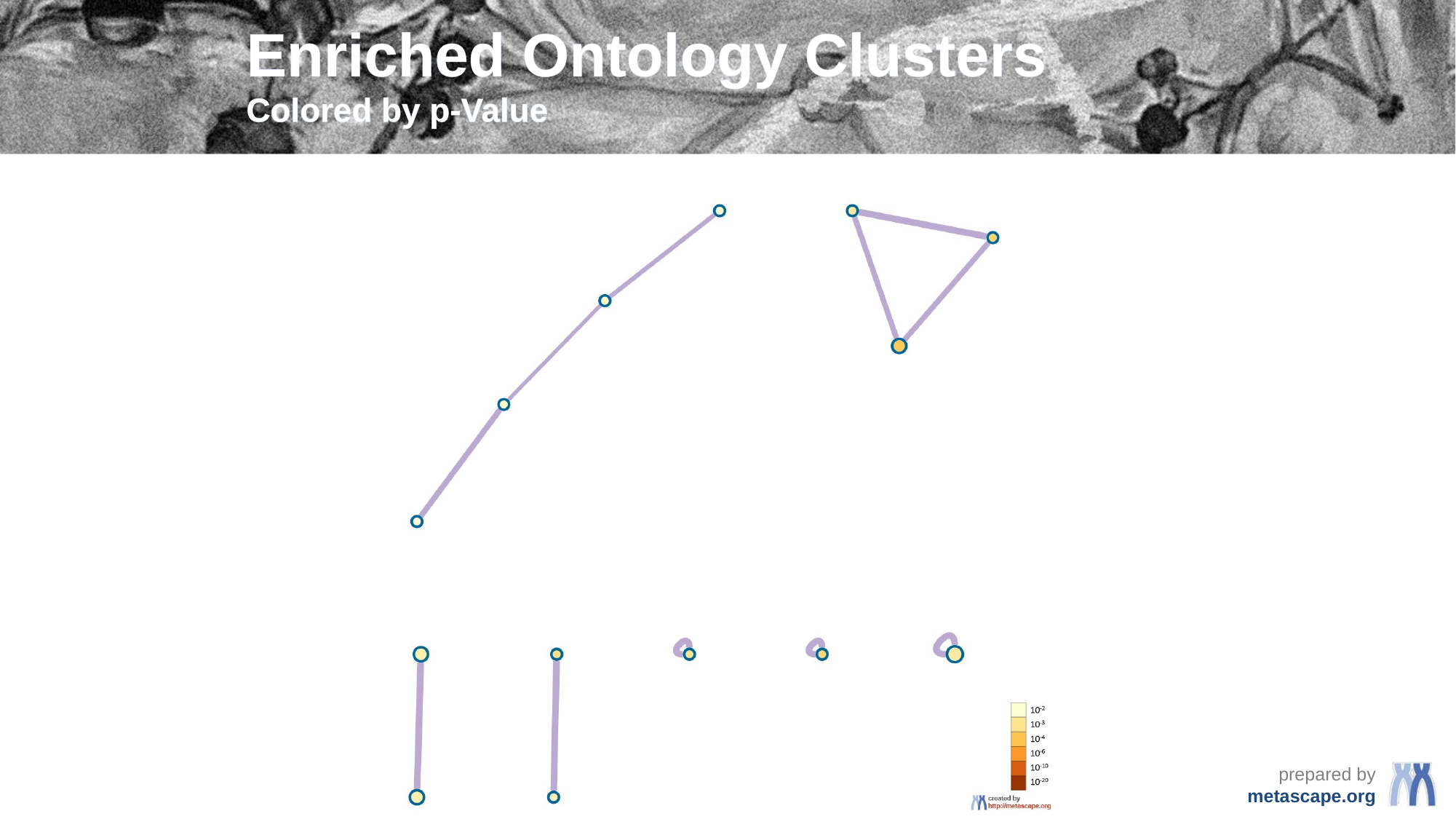

# Enriched Ontology Clusters Colored by p-Value

## Slide 6
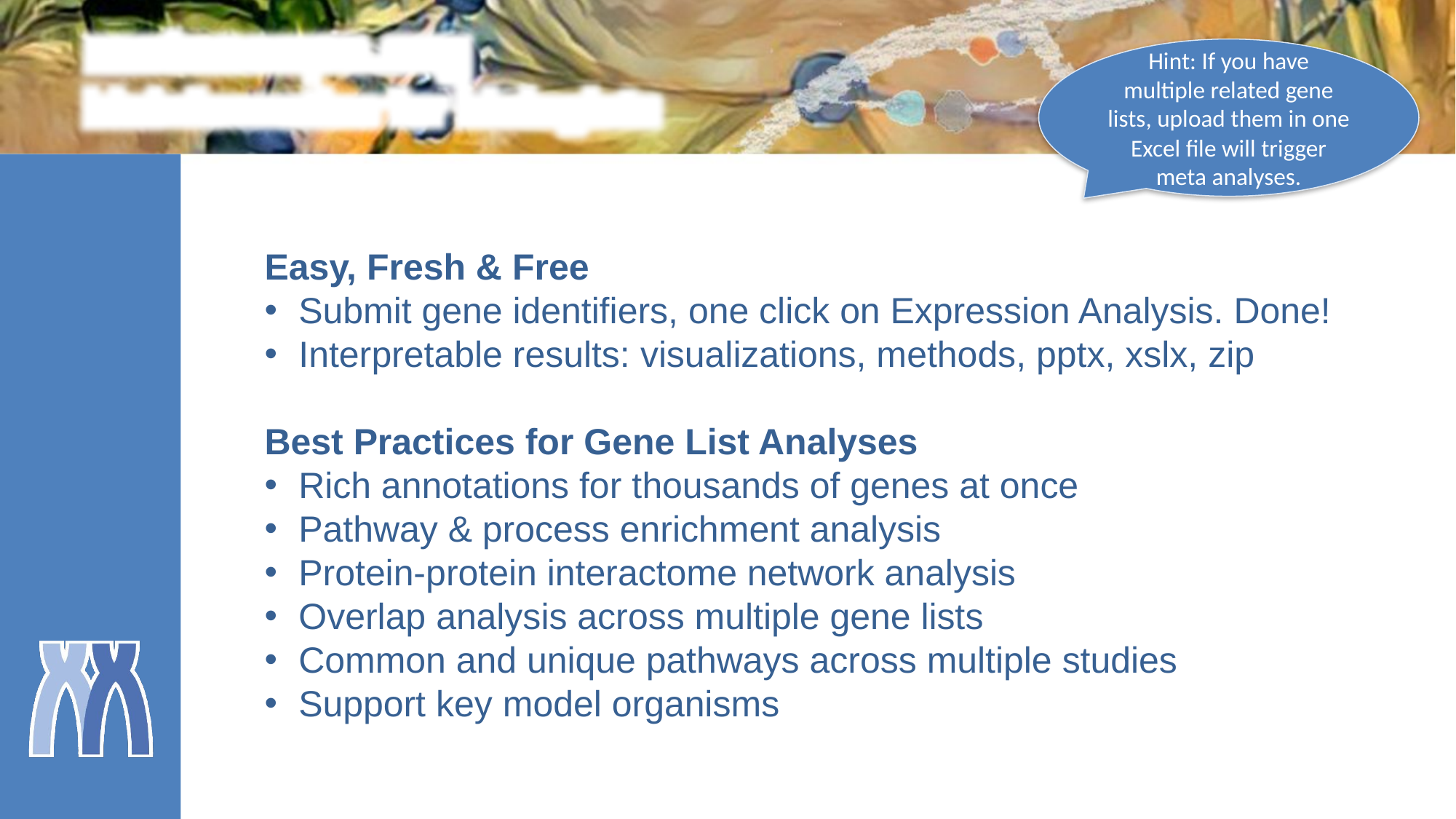

metascape.org
bioinformatics for biologists
Hint: If you have multiple related gene lists, upload them in one Excel file will trigger meta analyses.
Easy, Fresh & Free
Submit gene identifiers, one click on Expression Analysis. Done!
Interpretable results: visualizations, methods, pptx, xslx, zip
Best Practices for Gene List Analyses
Rich annotations for thousands of genes at once
Pathway & process enrichment analysis
Protein-protein interactome network analysis
Overlap analysis across multiple gene lists
Common and unique pathways across multiple studies
Support key model organisms
