# Supplementary material for: Bioinformatic Analyzes of the Association Between Upregulated Expression of JUN Gene via APOBEC-Induced FLG Gene Mutation and Prognosis of Cervical Cancer
Source: Front Med (Lausanne). 2022 Apr 18;9:815450. doi: 10.3389/fmed.2022.815450 (PMC9058067; doi:10.3389/fmed.2022.815450)
Supplement: Supplementary file 3 [file Data_Sheet_3.ZIP › Enrichment_GO/ColorByCluster.pdf]

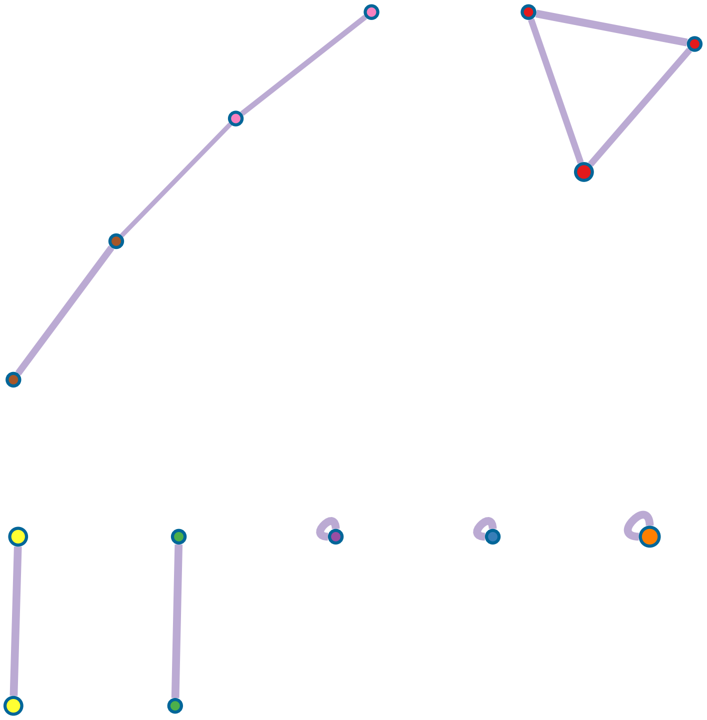

- glycosyltransferase activity
- calcium-dependent protein binding
- scavenger receptor activity
- protein kinase A binding
- signaling receptor regulator activity
- DNA-binding transcription activator activity, RNA poly
- phosphatidylinositol phosphate binding
- alcohol binding

created by
http://metascape.org
