# Supplementary material for: Bioinformatic Analyzes of the Association Between Upregulated Expression of JUN Gene via APOBEC-Induced FLG Gene Mutation and Prognosis of Cervical Cancer
Source: Front Med (Lausanne). 2022 Apr 18;9:815450. doi: 10.3389/fmed.2022.815450 (PMC9058067; doi:10.3389/fmed.2022.815450)
Supplement: Supplementary file 4 [file Data_Sheet_4.ZIP › Enrichment_GO/ColorByCluster.pdf]

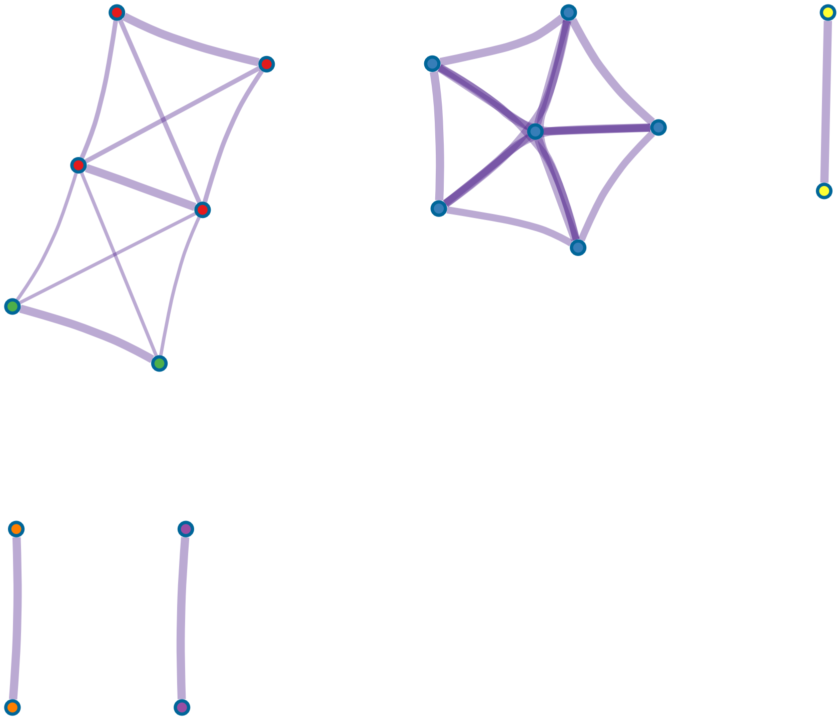

IL-17 signaling pathway
Chemical carcinogenesis
Endocrine resistance
Complement and coagulation cascades
Purine metabolism
Fc gamma R-mediated phagocytosis

created by
http://metascape.org
