# Supplementary material for: Bioinformatic Analyzes of the Association Between Upregulated Expression of JUN Gene via APOBEC-Induced FLG Gene Mutation and Prognosis of Cervical Cancer
Source: Front Med (Lausanne). 2022 Apr 18;9:815450. doi: 10.3389/fmed.2022.815450 (PMC9058067; doi:10.3389/fmed.2022.815450)
Supplement: Supplementary file 4 [file Data_Sheet_4.ZIP › AnalysisReport.html]

## Metascape Gene List Analysis Report

metascape.org1

### Bar Graph Summary

Figure 1. Bar graph of enriched terms across input gene lists, colored by p-values.

|  |
| --- |
|  |
|  |

### Gene Lists

User-provided gene identifiers are first converted into their corresponding H. sapiens Entrez gene IDs using the latest version of the database (last updated on 2021-11-01). If multiple identifiers correspond to the same Entrez gene ID, they will be considered as a single Entrez gene ID in downstream analyses. The gene lists are summarized in Table 1.

Table 1. Statistics of input gene lists.

| Name | Total | Unique |
| --- | --- | --- |
| MyList | 124 | 124 |

### Pathway and Process Enrichment Analysis

For each given gene list, pathway and process enrichment analysis has been carried out with the following ontology sources: KEGG Pathway. All genes in the genome have been used as the enrichment background. Terms with a p-value < 0.01, a minimum count of 3, and an enrichment factor > 1.5 (the enrichment factor is the ratio between the observed counts and the counts expected by chance) are collected and grouped into clusters based on their membership similarities. More specifically, p-values are calculated based on the accumulative hypergeometric distribution2, and q-values are calculated using the Banjamini-Hochberg procedure to account for multiple testings3. Kappa scores4 are used as the similarity metric when performing hierachical clustering on the enriched terms, and sub-trees with a similarity of > 0.3 are considered a cluster. The most statistically significant term within a cluster is chosen to represent the cluster.

Table 2. Top 6 clusters with their representative enriched terms (one per cluster). "Count" is the number of genes in the user-provided lists with membership in the given ontology term. "%" is the percentage of all of the user-provided genes that are found in the given ontology term (only input genes with at least one ontology term annotation are included in the calculation). "Log10(P)" is the p-value in log base 10. "Log10(q)" is the multi-test adjusted p-value in log base 10.

| GO | Category | Description | Count | % | Log10(P) | Log10(q) |
| --- | --- | --- | --- | --- | --- | --- |
| ko04657 | KEGG Pathway | IL-17 signaling pathway | 5 | 4.03 | -4.38 | -1.74 |
| ko05204 | KEGG Pathway | Chemical carcinogenesis | 4 | 3.23 | -3.43 | -1.23 |
| hsa01522 | KEGG Pathway | Endocrine resistance | 4 | 3.23 | -3.17 | -1.18 |
| ko04610 | KEGG Pathway | Complement and coagulation cascades | 3 | 2.42 | -2.37 | -0.59 |
| ko00230 | KEGG Pathway | Purine metabolism | 4 | 3.23 | -2.24 | -0.51 |
| ko04666 | KEGG Pathway | Fc gamma R-mediated phagocytosis | 3 | 2.42 | -2.20 | -0.51 |

To further capture the relationships between the terms, a subset of enriched terms have been selected and rendered as a network plot, where terms with a similarity > 0.3 are connected by edges. We select the terms with the best p-values from each of the 20 clusters, with the constraint that there are no more than 15 terms per cluster and no more than 250 terms in total. The network is visualized using Cytoscape5, where each node represents an enriched term and is colored first by its cluster ID (Figure 2.a) and then by its p-value (Figure 2.b). These networks can be interactively viewed in Cytoscape through the .cys files (contained in the Zip package, which also contains a publication-quality version as a PDF) or within a browser by clicking on the web icon. For clarity, term labels are only shown for one term per cluster, so it is recommended to use Cytoscape or a browser to visualize the network in order to inspect all node labels. We can also export the network into a PDF file within Cytoscape, and then edit the labels using Adobe Illustrator for publication purposes. To switch off all labels, delete the "Label" mapping under the "Style" tab within Cytoscape, and then export the network view.

Figure 2. Network of enriched terms: (a) colored by cluster ID, where nodes that share the same cluster ID are typically close to each other; (b) colored by p-value, where terms containing more genes tend to have a more significant p-value.

|  |  |
| --- | --- |
|  |  |
|  |  |

### Reference

1. Zhou et al., Metascape provides a biologist-oriented resource for the analysis of systems-level datasets. Nature Communications (2019) 10(1):1523.
2. Zar, J.H. Biostatistical Analysis 1999 4th edn., NJ Prentice Hall, pp. 523
3. Hochberg Y., Benjamini Y. More powerful procedures for multiple significance testing. Statistics in Medicine (1990) 9:811-818.
4. Cohen, J. A coefficient of agreement for nominal scales. Educ. Psychol. Meas. (1960) 20:27-46.
5. Shannon P. et al., Cytoscape: a software environment for integrated models of biomolecular interaction networks. Genome Res (2003) 11:2498-2504.
